# Probing lepton flavour violation via neutrinoless $\varvec{\tau \longrightarrow 3\mu }$ decays with the ATLAS detector

**DOI:** 10.1140/epjc/s10052-016-4041-9

**Published:** 2016-04-26

**Authors:** Atlas Collaboration, G. Aad, B. Abbott, J. Abdallah, O. Abdinov, R. Aben, M. Abolins, O. S. AbouZeid, H. Abramowicz, H. Abreu, R. Abreu, Y. Abulaiti, B. S. Acharya, L. Adamczyk, D. L. Adams, J. Adelman, S. Adomeit, T. Adye, A. A. Affolder, T. Agatonovic-Jovin, J. Agricola, J. A. Aguilar-Saavedra, S. P. Ahlen, F. Ahmadov, G. Aielli, H. Akerstedt, T. P. A. Åkesson, A. V. Akimov, G. L. Alberghi, J. Albert, S. Albrand, M. J. Alconada Verzini, M. Aleksa, I. N. Aleksandrov, C. Alexa, G. Alexander, T. Alexopoulos, M. Alhroob, G. Alimonti, L. Alio, J. Alison, S. P. Alkire, B. M. M. Allbrooke, P. P. Allport, A. Aloisio, A. Alonso, F. Alonso, C. Alpigiani, A. Altheimer, B. Alvarez Gonzalez, D. Álvarez Piqueras, M. G. Alviggi, B. T. Amadio, K. Amako, Y. Amaral Coutinho, C. Amelung, D. Amidei, S. P. Amor Dos Santos, A. Amorim, S. Amoroso, N. Amram, G. Amundsen, C. Anastopoulos, L. S. Ancu, N. Andari, T. Andeen, C. F. Anders, G. Anders, J. K. Anders, K. J. Anderson, A. Andreazza, V. Andrei, S. Angelidakis, I. Angelozzi, P. Anger, A. Angerami, F. Anghinolfi, A. V. Anisenkov, N. Anjos, A. Annovi, M. Antonelli, A. Antonov, J. Antos, F. Anulli, M. Aoki, L. Aperio Bella, G. Arabidze, Y. Arai, J. P. Araque, A. T. H. Arce, F. A. Arduh, J-F. Arguin, S. Argyropoulos, M. Arik, A. J. Armbruster, O. Arnaez, H. Arnold, M. Arratia, O. Arslan, A. Artamonov, G. Artoni, S. Asai, N. Asbah, A. Ashkenazi, B. Åsman, L. Asquith, K. Assamagan, R. Astalos, M. Atkinson, N. B. Atlay, K. Augsten, M. Aurousseau, G. Avolio, B. Axen, M. K. Ayoub, G. Azuelos, M. A. Baak, A. E. Baas, M. J. Baca, C. Bacci, H. Bachacou, K. Bachas, M. Backes, M. Backhaus, P. Bagiacchi, P. Bagnaia, Y. Bai, T. Bain, J. T. Baines, O. K. Baker, E. M. Baldin, P. Balek, T. Balestri, F. Balli, W. K. Balunas, E. Banas, Sw. Banerjee, A. A. E. Bannoura, L. Barak, E. L. Barberio, D. Barberis, M. Barbero, T. Barillari, M. Barisonzi, T. Barklow, N. Barlow, S. L. Barnes, B. M. Barnett, R. M. Barnett, Z. Barnovska, A. Baroncelli, G. Barone, A. J. Barr, F. Barreiro, J. Barreiro Guimarães da Costa, R. Bartoldus, A. E. Barton, P. Bartos, A. Basalaev, A. Bassalat, A. Basye, R. L. Bates, S. J. Batista, J. R. Batley, M. Battaglia, M. Bauce, F. Bauer, H. S. Bawa, J. B. Beacham, M. D. Beattie, T. Beau, P. H. Beauchemin, R. Beccherle, P. Bechtle, H. P. Beck, K. Becker, M. Becker, M. Beckingham, C. Becot, A. J. Beddall, A. Beddall, V. A. Bednyakov, C. P. Bee, L. J. Beemster, T. A. Beermann, M. Begel, J. K. Behr, C. Belanger-Champagne, W. H. Bell, G. Bella, L. Bellagamba, A. Bellerive, M. Bellomo, K. Belotskiy, O. Beltramello, O. Benary, D. Benchekroun, M. Bender, K. Bendtz, N. Benekos, Y. Benhammou, E. Benhar Noccioli, J. A. Benitez Garcia, D. P. Benjamin, J. R. Bensinger, S. Bentvelsen, L. Beresford, M. Beretta, D. Berge, E. Bergeaas Kuutmann, N. Berger, F. Berghaus, J. Beringer, C. Bernard, N. R. Bernard, C. Bernius, F. U. Bernlochner, T. Berry, P. Berta, C. Bertella, G. Bertoli, F. Bertolucci, C. Bertsche, D. Bertsche, M. I. Besana, G. J. Besjes, O. Bessidskaia Bylund, M. Bessner, N. Besson, C. Betancourt, S. Bethke, A. J. Bevan, W. Bhimji, R. M. Bianchi, L. Bianchini, M. Bianco, O. Biebel, D. Biedermann, S. P. Bieniek, N. V. Biesuz, M. Biglietti, J. Bilbao De Mendizabal, H. Bilokon, M. Bindi, S. Binet, A. Bingul, C. Bini, S. Biondi, D. M. Bjergaard, C. W. Black, J. E. Black, K. M. Black, D. Blackburn, R. E. Blair, J.-B. Blanchard, J. E. Blanco, T. Blazek, I. Bloch, C. Blocker, W. Blum, U. Blumenschein, S. Blunier, G. J. Bobbink, V. S. Bobrovnikov, S. S. Bocchetta, A. Bocci, C. Bock, M. Boehler, J. A. Bogaerts, D. Bogavac, A. G. Bogdanchikov, C. Bohm, V. Boisvert, T. Bold, V. Boldea, A. S. Boldyrev, M. Bomben, M. Bona, M. Boonekamp, A. Borisov, G. Borissov, S. Borroni, J. Bortfeldt, V. Bortolotto, K. Bos, D. Boscherini, M. Bosman, J. Boudreau, J. Bouffard, E. V. Bouhova-Thacker, D. Boumediene, C. Bourdarios, N. Bousson, S. K. Boutle, A. Boveia, J. Boyd, I. R. Boyko, I. Bozic, J. Bracinik, A. Brandt, G. Brandt, O. Brandt, U. Bratzler, B. Brau, J. E. Brau, H. M. Braun, W. D. Breaden Madden, K. Brendlinger, A. J. Brennan, L. Brenner, R. Brenner, S. Bressler, T. M. Bristow, D. Britton, D. Britzger, F. M. Brochu, I. Brock, R. Brock, J. Bronner, G. Brooijmans, T. Brooks, W. K. Brooks, J. Brosamer, E. Brost, P. A. Bruckman de Renstrom, D. Bruncko, R. Bruneliere, A. Bruni, G. Bruni, M. Bruschi, N. Bruscino, L. Bryngemark, T. Buanes, Q. Buat, P. Buchholz, A. G. Buckley, S. I. Buda, I. A. Budagov, F. Buehrer, L. Bugge, M. K. Bugge, O. Bulekov, D. Bullock, H. Burckhart, S. Burdin, C. D. Burgard, B. Burghgrave, S. Burke, I. Burmeister, E. Busato, D. Büscher, V. Büscher, P. Bussey, J. M. Butler, A. I. Butt, C. M. Buttar, J. M. Butterworth, P. Butti, W. Buttinger, A. Buzatu, A. R. Buzykaev, S. Cabrera Urbán, D. Caforio, V. M. Cairo, O. Cakir, N. Calace, P. Calafiura, A. Calandri, G. Calderini, P. Calfayan, L. P. Caloba, D. Calvet, S. Calvet, R. Camacho Toro, S. Camarda, P. Camarri, D. Cameron, R. Caminal Armadans, S. Campana, M. Campanelli, A. Campoverde, V. Canale, A. Canepa, M. Cano Bret, J. Cantero, R. Cantrill, T. Cao, M. D. M. Capeans Garrido, I. Caprini, M. Caprini, M. Capua, R. Caputo, R. M. Carbone, R. Cardarelli, F. Cardillo, T. Carli, G. Carlino, L. Carminati, S. Caron, E. Carquin, G. D. Carrillo-Montoya, J. R. Carter, J. Carvalho, D. Casadei, M. P. Casado, M. Casolino, E. Castaneda-Miranda, A. Castelli, V. Castillo Gimenez, N. F. Castro, P. Catastini, A. Catinaccio, J. R. Catmore, A. Cattai, J. Caudron, V. Cavaliere, D. Cavalli, M. Cavalli-Sforza, V. Cavasinni, F. Ceradini, B. C. Cerio, K. Cerny, A. S. Cerqueira, A. Cerri, L. Cerrito, F. Cerutti, M. Cerv, A. Cervelli, S. A. Cetin, A. Chafaq, D. Chakraborty, I. Chalupkova, Y. L. Chan, P. Chang, J. D. Chapman, D. G. Charlton, C. C. Chau, C. A. Chavez Barajas, S. Cheatham, A. Chegwidden, S. Chekanov, S. V. Chekulaev, G. A. Chelkov, M. A. Chelstowska, C. Chen, H. Chen, K. Chen, L. Chen, S. Chen, S. Chen, X. Chen, Y. Chen, H. C. Cheng, Y. Cheng, A. Cheplakov, E. Cheremushkina, R. Cherkaoui El Moursli, V. Chernyatin, E. Cheu, L. Chevalier, V. Chiarella, G. Chiarelli, G. Chiodini, A. S. Chisholm, R. T. Chislett, A. Chitan, M. V. Chizhov, K. Choi, S. Chouridou, B. K. B. Chow, V. Christodoulou, D. Chromek-Burckhart, J. Chudoba, A. J. Chuinard, J. J. Chwastowski, L. Chytka, G. Ciapetti, A. K. Ciftci, D. Cinca, V. Cindro, I. A. Cioara, A. Ciocio, F. Cirotto, Z. H. Citron, M. Ciubancan, A. Clark, B. L. Clark, P. J. Clark, R. N. Clarke, C. Clement, Y. Coadou, M. Cobal, A. Coccaro, J. Cochran, L. Coffey, J. G. Cogan, L. Colasurdo, B. Cole, S. Cole, A. P. Colijn, J. Collot, T. Colombo, G. Compostella, P. Conde Muiño, E. Coniavitis, S. H. Connell, I. A. Connelly, V. Consorti, S. Constantinescu, C. Conta, G. Conti, F. Conventi, M. Cooke, B. D. Cooper, A. M. Cooper-Sarkar, T. Cornelissen, M. Corradi, F. Corriveau, A. Corso-Radu, A. Cortes-Gonzalez, G. Cortiana, G. Costa, M. J. Costa, D. Costanzo, D. Côté, G. Cottin, G. Cowan, B. E. Cox, K. Cranmer, G. Cree, S. Crépé-Renaudin, F. Crescioli, W. A. Cribbs, M. Crispin Ortuzar, M. Cristinziani, V. Croft, G. Crosetti, T. Cuhadar Donszelmann, J. Cummings, M. Curatolo, J. Cúth, C. Cuthbert, H. Czirr, P. Czodrowski, S. D’Auria, M. D’Onofrio, M. J. Da Cunha Sargedas De Sousa, C. Da Via, W. Dabrowski, A. Dafinca, T. Dai, O. Dale, F. Dallaire, C. Dallapiccola, M. Dam, J. R. Dandoy, N. P. Dang, A. C. Daniells, M. Danninger, M. Dano Hoffmann, V. Dao, G. Darbo, S. Darmora, J. Dassoulas, A. Dattagupta, W. Davey, C. David, T. Davidek, E. Davies, M. Davies, P. Davison, Y. Davygora, E. Dawe, I. Dawson, R. K. Daya-Ishmukhametova, K. De, R. de Asmundis, A. De Benedetti, S. De Castro, S. De Cecco, N. De Groot, P. de Jong, H. De la Torre, F. De Lorenzi, D. De Pedis, A. De Salvo, U. De Sanctis, A. De Santo, J. B. De Vivie De Regie, W. J. Dearnaley, R. Debbe, C. Debenedetti, D. V. Dedovich, I. Deigaard, J. Del Peso, T. Del Prete, D. Delgove, F. Deliot, C. M. Delitzsch, M. Deliyergiyev, A. Dell’Acqua, L. Dell’Asta, M. Dell’Orso, M. Della Pietra, D. della Volpe, M. Delmastro, P. A. Delsart, C. Deluca, D. A. DeMarco, S. Demers, M. Demichev, A. Demilly, S. P. Denisov, D. Derendarz, J. E. Derkaoui, F. Derue, P. Dervan, K. Desch, C. Deterre, K. Dette, P. O. Deviveiros, A. Dewhurst, S. Dhaliwal, A. Di Ciaccio, L. Di Ciaccio, A. Di Domenico, C. Di Donato, A. Di Girolamo, B. Di Girolamo, A. Di Mattia, B. Di Micco, R. Di Nardo, A. Di Simone, R. Di Sipio, D. Di Valentino, C. Diaconu, M. Diamond, F. A. Dias, M. A. Diaz, E. B. Diehl, J. Dietrich, S. Diglio, A. Dimitrievska, J. Dingfelder, P. Dita, S. Dita, F. Dittus, F. Djama, T. Djobava, J. I. Djuvsland, M. A. B. do Vale, D. Dobos, M. Dobre, C. Doglioni, T. Dohmae, J. Dolejsi, Z. Dolezal, B. A. Dolgoshein, M. Donadelli, S. Donati, P. Dondero, J. Donini, J. Dopke, A. Doria, M. T. Dova, A. T. Doyle, E. Drechsler, M. Dris, E. Dubreuil, E. Duchovni, G. Duckeck, O. A. Ducu, D. Duda, A. Dudarev, L. Duflot, L. Duguid, M. Dührssen, M. Dunford, H. Duran Yildiz, M. Düren, A. Durglishvili, D. Duschinger, B. Dutta, M. Dyndal, C. Eckardt, K. M. Ecker, R. C. Edgar, W. Edson, N. C. Edwards, W. Ehrenfeld, T. Eifert, G. Eigen, K. Einsweiler, T. Ekelof, M. El Kacimi, M. Ellert, S. Elles, F. Ellinghaus, A. A. Elliot, N. Ellis, J. Elmsheuser, M. Elsing, D. Emeliyanov, Y. Enari, O. C. Endner, M. Endo, J. Erdmann, A. Ereditato, G. Ernis, J. Ernst, M. Ernst, S. Errede, E. Ertel, M. Escalier, H. Esch, C. Escobar, B. Esposito, A. I. Etienvre, E. Etzion, H. Evans, A. Ezhilov, L. Fabbri, G. Facini, R. M. Fakhrutdinov, S. Falciano, R. J. Falla, J. Faltova, Y. Fang, M. Fanti, A. Farbin, A. Farilla, T. Farooque, S. Farrell, S. M. Farrington, P. Farthouat, F. Fassi, P. Fassnacht, D. Fassouliotis, M. Faucci Giannelli, A. Favareto, L. Fayard, O. L. Fedin, W. Fedorko, S. Feigl, L. Feligioni, C. Feng, E. J. Feng, H. Feng, A. B. Fenyuk, L. Feremenga, P. Fernandez Martinez, S. Fernandez Perez, J. Ferrando, A. Ferrari, P. Ferrari, R. Ferrari, D. E. Ferreira de Lima, A. Ferrer, D. Ferrere, C. Ferretti, A. Ferretto Parodi, M. Fiascaris, F. Fiedler, A. Filipčič, M. Filipuzzi, F. Filthaut, M. Fincke-Keeler, K. D. Finelli, M. C. N. Fiolhais, L. Fiorini, A. Firan, A. Fischer, C. Fischer, J. Fischer, W. C. Fisher, N. Flaschel, I. Fleck, P. Fleischmann, G. T. Fletcher, G. Fletcher, R. R. M. Fletcher, T. Flick, A. Floderus, L. R. Flores Castillo, M. J. Flowerdew, A. Formica, A. Forti, D. Fournier, H. Fox, S. Fracchia, P. Francavilla, M. Franchini, D. Francis, L. Franconi, M. Franklin, M. Frate, M. Fraternali, D. Freeborn, S. T. French, F. Friedrich, D. Froidevaux, J. A. Frost, C. Fukunaga, E. Fullana Torregrosa, B. G. Fulsom, T. Fusayasu, J. Fuster, C. Gabaldon, O. Gabizon, A. Gabrielli, A. Gabrielli, G. P. Gach, S. Gadatsch, S. Gadomski, G. Gagliardi, P. Gagnon, C. Galea, B. Galhardo, E. J. Gallas, B. J. Gallop, P. Gallus, G. Galster, K. K. Gan, J. Gao, Y. Gao, Y. S. Gao, F. M. Garay Walls, F. Garberson, C. García, J. E. García Navarro, M. Garcia-Sciveres, R. W. Gardner, N. Garelli, V. Garonne, C. Gatti, A. Gaudiello, G. Gaudio, B. Gaur, L. Gauthier, P. Gauzzi, I. L. Gavrilenko, C. Gay, G. Gaycken, E. N. Gazis, P. Ge, Z. Gecse, C. N. P. Gee, Ch. Geich-Gimbel, M. P. Geisler, C. Gemme, M. H. Genest, S. Gentile, M. George, S. George, D. Gerbaudo, A. Gershon, S. Ghasemi, H. Ghazlane, B. Giacobbe, S. Giagu, V. Giangiobbe, P. Giannetti, B. Gibbard, S. M. Gibson, M. Gignac, M. Gilchriese, T. P. S. Gillam, D. Gillberg, G. Gilles, D. M. Gingrich, N. Giokaris, M. P. Giordani, F. M. Giorgi, F. M. Giorgi, P. F. Giraud, P. Giromini, D. Giugni, C. Giuliani, M. Giulini, B. K. Gjelsten, S. Gkaitatzis, I. Gkialas, E. L. Gkougkousis, L. K. Gladilin, C. Glasman, J. Glatzer, P. C. F. Glaysher, A. Glazov, M. Goblirsch-Kolb, J. R. Goddard, J. Godlewski, S. Goldfarb, T. Golling, D. Golubkov, A. Gomes, R. Gonçalo, J. Goncalves Pinto Firmino Da Costa, L. Gonella, S. González de la Hoz, G. Gonzalez Parra, S. Gonzalez-Sevilla, L. Goossens, P. A. Gorbounov, H. A. Gordon, I. Gorelov, B. Gorini, E. Gorini, A. Gorišek, E. Gornicki, A. T. Goshaw, C. Gössling, M. I. Gostkin, D. Goujdami, A. G. Goussiou, N. Govender, E. Gozani, H. M. X. Grabas, L. Graber, I. Grabowska-Bold, P. O. J. Gradin, P. Grafström, J. Gramling, E. Gramstad, S. Grancagnolo, V. Gratchev, H. M. Gray, E. Graziani, Z. D. Greenwood, C. Grefe, K. Gregersen, I. M. Gregor, P. Grenier, J. Griffiths, A. A. Grillo, K. Grimm, S. Grinstein, Ph. Gris, J.-F. Grivaz, J. P. Grohs, A. Grohsjean, E. Gross, J. Grosse-Knetter, G. C. Grossi, Z. J. Grout, L. Guan, J. Guenther, F. Guescini, D. Guest, O. Gueta, E. Guido, T. Guillemin, S. Guindon, U. Gul, C. Gumpert, J. Guo, Y. Guo, S. Gupta, G. Gustavino, P. Gutierrez, N. G. Gutierrez Ortiz, C. Gutschow, C. Guyot, C. Gwenlan, C. B. Gwilliam, A. Haas, C. Haber, H. K. Hadavand, N. Haddad, P. Haefner, S. Hageböck, Z. Hajduk, H. Hakobyan, M. Haleem, J. Haley, D. Hall, G. Halladjian, G. D. Hallewell, K. Hamacher, P. Hamal, K. Hamano, A. Hamilton, G. N. Hamity, P. G. Hamnett, L. Han, K. Hanagaki, K. Hanawa, M. Hance, B. Haney, P. Hanke, R. Hanna, J. B. Hansen, J. D. Hansen, M. C. Hansen, P. H. Hansen, K. Hara, A. S. Hard, T. Harenberg, F. Hariri, S. Harkusha, R. D. Harrington, P. F. Harrison, F. Hartjes, M. Hasegawa, Y. Hasegawa, A. Hasib, S. Hassani, S. Haug, R. Hauser, L. Hauswald, M. Havranek, C. M. Hawkes, R. J. Hawkings, A. D. Hawkins, T. Hayashi, D. Hayden, C. P. Hays, J. M. Hays, H. S. Hayward, S. J. Haywood, S. J. Head, T. Heck, V. Hedberg, L. Heelan, S. Heim, T. Heim, B. Heinemann, L. Heinrich, J. Hejbal, L. Helary, S. Hellman, D. Hellmich, C. Helsens, J. Henderson, R. C. W. Henderson, Y. Heng, C. Hengler, S. Henkelmann, A. Henrichs, A. M. Henriques Correia, S. Henrot-Versille, G. H. Herbert, Y. Hernández Jiménez, G. Herten, R. Hertenberger, L. Hervas, G. G. Hesketh, N. P. Hessey, J. W. Hetherly, R. Hickling, E. Higón-Rodriguez, E. Hill, J. C. Hill, K. H. Hiller, S. J. Hillier, I. Hinchliffe, E. Hines, R. R. Hinman, M. Hirose, D. Hirschbuehl, J. Hobbs, N. Hod, M. C. Hodgkinson, P. Hodgson, A. Hoecker, M. R. Hoeferkamp, F. Hoenig, M. Hohlfeld, D. Hohn, T. R. Holmes, M. Homann, T. M. Hong, W. H. Hopkins, Y. Horii, A. J. Horton, J-Y. Hostachy, S. Hou, A. Hoummada, J. Howard, J. Howarth, M. Hrabovsky, I. Hristova, J. Hrivnac, T. Hryn’ova, A. Hrynevich, C. Hsu, P. J. Hsu, S.-C. Hsu, D. Hu, Q. Hu, X. Hu, Y. Huang, Z. Hubacek, F. Hubaut, F. Huegging, T. B. Huffman, E. W. Hughes, G. Hughes, M. Huhtinen, T. A. Hülsing, N. Huseynov, J. Huston, J. Huth, G. Iacobucci, G. Iakovidis, I. Ibragimov, L. Iconomidou-Fayard, E. Ideal, Z. Idrissi, P. Iengo, O. Igonkina, T. Iizawa, Y. Ikegami, M. Ikeno, Y. Ilchenko, D. Iliadis, N. Ilic, T. Ince, G. Introzzi, P. Ioannou, M. Iodice, K. Iordanidou, V. Ippolito, A. Irles Quiles, C. Isaksson, M. Ishino, M. Ishitsuka, R. Ishmukhametov, C. Issever, S. Istin, J. M. Iturbe Ponce, R. Iuppa, J. Ivarsson, W. Iwanski, H. Iwasaki, J. M. Izen, V. Izzo, S. Jabbar, B. Jackson, M. Jackson, P. Jackson, M. R. Jaekel, V. Jain, K. Jakobs, S. Jakobsen, T. Jakoubek, J. Jakubek, D. O. Jamin, D. K. Jana, E. Jansen, R. Jansky, J. Janssen, M. Janus, G. Jarlskog, N. Javadov, T. Javůrek, L. Jeanty, J. Jejelava, G.-Y. Jeng, D. Jennens, P. Jenni, J. Jentzsch, C. Jeske, S. Jézéquel, H. Ji, J. Jia, Y. Jiang, S. Jiggins, J. Jimenez Pena, S. Jin, A. Jinaru, O. Jinnouchi, M. D. Joergensen, P. Johansson, K. A. Johns, W. J. Johnson, K. Jon-And, G. Jones, R. W. L. Jones, T. J. Jones, J. Jongmanns, P. M. Jorge, K. D. Joshi, J. Jovicevic, X. Ju, P. Jussel, A. Juste Rozas, M. Kaci, A. Kaczmarska, M. Kado, H. Kagan, M. Kagan, S. J. Kahn, E. Kajomovitz, C. W. Kalderon, S. Kama, A. Kamenshchikov, N. Kanaya, S. Kaneti, V. A. Kantserov, J. Kanzaki, B. Kaplan, L. S. Kaplan, A. Kapliy, D. Kar, K. Karakostas, A. Karamaoun, N. Karastathis, M. J. Kareem, E. Karentzos, M. Karnevskiy, S. N. Karpov, Z. M. Karpova, K. Karthik, V. Kartvelishvili, A. N. Karyukhin, K. Kasahara, L. Kashif, R. D. Kass, A. Kastanas, Y. Kataoka, C. Kato, A. Katre, J. Katzy, K. Kawade, K. Kawagoe, T. Kawamoto, G. Kawamura, S. Kazama, V. F. Kazanin, R. Keeler, R. Kehoe, J. S. Keller, J. J. Kempster, H. Keoshkerian, O. Kepka, B. P. Kerševan, S. Kersten, R. A. Keyes, F. Khalil-zada, H. Khandanyan, A. Khanov, A. G. Kharlamov, T. J. Khoo, V. Khovanskiy, E. Khramov, J. Khubua, S. Kido, H. Y. Kim, S. H. Kim, Y. K. Kim, N. Kimura, O. M. Kind, B. T. King, M. King, S. B. King, J. Kirk, A. E. Kiryunin, T. Kishimoto, D. Kisielewska, F. Kiss, K. Kiuchi, O. Kivernyk, E. Kladiva, M. H. Klein, M. Klein, U. Klein, K. Kleinknecht, P. Klimek, A. Klimentov, R. Klingenberg, J. A. Klinger, T. Klioutchnikova, E.-E. Kluge, P. Kluit, S. Kluth, J. Knapik, E. Kneringer, E. B. F. G. Knoops, A. Knue, A. Kobayashi, D. Kobayashi, T. Kobayashi, M. Kobel, M. Kocian, P. Kodys, T. Koffas, E. Koffeman, L. A. Kogan, S. Kohlmann, Z. Kohout, T. Kohriki, T. Koi, H. Kolanoski, M. Kolb, I. Koletsou, A. A. Komar, Y. Komori, T. Kondo, N. Kondrashova, K. Köneke, A. C. König, T. Kono, R. Konoplich, N. Konstantinidis, R. Kopeliansky, S. Koperny, L. Köpke, A. K. Kopp, K. Korcyl, K. Kordas, A. Korn, A. A. Korol, I. Korolkov, E. V. Korolkova, O. Kortner, S. Kortner, T. Kosek, V. V. Kostyukhin, V. M. Kotov, A. Kotwal, A. Kourkoumeli-Charalampidi, C. Kourkoumelis, V. Kouskoura, A. Koutsman, R. Kowalewski, T. Z. Kowalski, W. Kozanecki, A. S. Kozhin, V. A. Kramarenko, G. Kramberger, D. Krasnopevtsev, M. W. Krasny, A. Krasznahorkay, J. K. Kraus, A. Kravchenko, S. Kreiss, M. Kretz, J. Kretzschmar, K. Kreutzfeldt, P. Krieger, K. Krizka, K. Kroeninger, H. Kroha, J. Kroll, J. Kroseberg, J. Krstic, U. Kruchonak, H. Krüger, N. Krumnack, A. Kruse, M. C. Kruse, M. Kruskal, T. Kubota, H. Kucuk, S. Kuday, S. Kuehn, A. Kugel, F. Kuger, A. Kuhl, T. Kuhl, V. Kukhtin, R. Kukla, Y. Kulchitsky, S. Kuleshov, M. Kuna, T. Kunigo, A. Kupco, H. Kurashige, Y. A. Kurochkin, V. Kus, E. S. Kuwertz, M. Kuze, J. Kvita, T. Kwan, D. Kyriazopoulos, A. La Rosa, J. L. La Rosa Navarro, L. La Rotonda, C. Lacasta, F. Lacava, J. Lacey, H. Lacker, D. Lacour, V. R. Lacuesta, E. Ladygin, R. Lafaye, B. Laforge, T. Lagouri, S. Lai, L. Lambourne, S. Lammers, C. L. Lampen, W. Lampl, E. Lançon, U. Landgraf, M. P. J. Landon, V. S. Lang, J. C. Lange, A. J. Lankford, F. Lanni, K. Lantzsch, A. Lanza, S. Laplace, C. Lapoire, J. F. Laporte, T. Lari, F. Lasagni Manghi, M. Lassnig, P. Laurelli, W. Lavrijsen, A. T. Law, P. Laycock, T. Lazovich, O. Le Dortz, E. Le Guirriec, E. Le Menedeu, M. LeBlanc, T. LeCompte, F. Ledroit-Guillon, C. A. Lee, S. C. Lee, L. Lee, G. Lefebvre, M. Lefebvre, F. Legger, C. Leggett, A. Lehan, G. Lehmann Miotto, X. Lei, W. A. Leight, A. Leisos, A. G. Leister, M. A. L. Leite, R. Leitner, D. Lellouch, B. Lemmer, K. J. C. Leney, T. Lenz, B. Lenzi, R. Leone, S. Leone, C. Leonidopoulos, S. Leontsinis, C. Leroy, C. G. Lester, M. Levchenko, J. Levêque, D. Levin, L. J. Levinson, M. Levy, A. Lewis, A. M. Leyko, M. Leyton, B. Li, H. Li, H. L. Li, L. Li, L. Li, S. Li, X. Li, Y. Li, Z. Liang, H. Liao, B. Liberti, A. Liblong, P. Lichard, K. Lie, J. Liebal, W. Liebig, C. Limbach, A. Limosani, S. C. Lin, T. H. Lin, F. Linde, B. E. Lindquist, J. T. Linnemann, E. Lipeles, A. Lipniacka, M. Lisovyi, T. M. Liss, D. Lissauer, A. Lister, A. M. Litke, B. Liu, D. Liu, H. Liu, J. Liu, J. B. Liu, K. Liu, L. Liu, M. Liu, M. Liu, Y. Liu, M. Livan, A. Lleres, J. Llorente Merino, S. L. Lloyd, F. Lo Sterzo, E. Lobodzinska, P. Loch, W. S. Lockman, F. K. Loebinger, A. E. Loevschall-Jensen, K. M. Loew, A. Loginov, T. Lohse, K. Lohwasser, M. Lokajicek, B. A. Long, J. D. Long, R. E. Long, K. A. Looper, L. Lopes, D. Lopez Mateos, B. Lopez Paredes, I. Lopez Paz, J. Lorenz, N. Lorenzo Martinez, M. Losada, P. J. Lösel, X. Lou, A. Lounis, J. Love, P. A. Love, H. Lu, N. Lu, H. J. Lubatti, C. Luci, A. Lucotte, C. Luedtke, F. Luehring, W. Lukas, L. Luminari, O. Lundberg, B. Lund-Jensen, D. Lynn, R. Lysak, E. Lytken, H. Ma, L. L. Ma, G. Maccarrone, A. Macchiolo, C. M. Macdonald, B. Maček, J. Machado Miguens, D. Macina, D. Madaffari, R. Madar, H. J. Maddocks, W. F. Mader, A. Madsen, J. Maeda, S. Maeland, T. Maeno, A. Maevskiy, E. Magradze, K. Mahboubi, J. Mahlstedt, C. Maiani, C. Maidantchik, A. A. Maier, T. Maier, A. Maio, S. Majewski, Y. Makida, N. Makovec, B. Malaescu, Pa. Malecki, V. P. Maleev, F. Malek, U. Mallik, D. Malon, C. Malone, S. Maltezos, V. M. Malyshev, S. Malyukov, J. Mamuzic, G. Mancini, B. Mandelli, L. Mandelli, I. Mandić, R. Mandrysch, J. Maneira, A. Manfredini, L. Manhaes de Andrade Filho, J. Manjarres Ramos, A. Mann, A. Manousakis-Katsikakis, B. Mansoulie, R. Mantifel, M. Mantoani, L. Mapelli, L. March, G. Marchiori, M. Marcisovsky, C. P. Marino, M. Marjanovic, D. E. Marley, F. Marroquim, S. P. Marsden, Z. Marshall, L. F. Marti, S. Marti-Garcia, B. Martin, T. A. Martin, V. J. Martin, B. Martin dit Latour, M. Martinez, S. Martin-Haugh, V. S. Martoiu, A. C. Martyniuk, M. Marx, F. Marzano, A. Marzin, L. Masetti, T. Mashimo, R. Mashinistov, J. Masik, A. L. Maslennikov, I. Massa, L. Massa, P. Mastrandrea, A. Mastroberardino, T. Masubuchi, P. Mättig, J. Mattmann, J. Maurer, S. J. Maxfield, D. A. Maximov, R. Mazini, S. M. Mazza, G. Mc Goldrick, S. P. Mc Kee, A. McCarn, R. L. McCarthy, T. G. McCarthy, N. A. McCubbin, K. W. McFarlane, J. A. Mcfayden, G. Mchedlidze, S. J. McMahon, R. A. McPherson, M. Medinnis, S. Meehan, S. Mehlhase, A. Mehta, K. Meier, C. Meineck, B. Meirose, B. R. Mellado Garcia, F. Meloni, A. Mengarelli, S. Menke, E. Meoni, K. M. Mercurio, S. Mergelmeyer, P. Mermod, L. Merola, C. Meroni, F. S. Merritt, A. Messina, J. Metcalfe, A. S. Mete, C. Meyer, C. Meyer, J-P. Meyer, J. Meyer, H. Meyer Zu Theenhausen, R. P. Middleton, S. Miglioranzi, L. Mijović, G. Mikenberg, M. Mikestikova, M. Mikuž, M. Milesi, A. Milic, D. W. Miller, C. Mills, A. Milov, D. A. Milstead, A. A. Minaenko, Y. Minami, I. A. Minashvili, A. I. Mincer, B. Mindur, M. Mineev, Y. Ming, L. M. Mir, K. P. Mistry, T. Mitani, J. Mitrevski, V. A. Mitsou, A. Miucci, P. S. Miyagawa, J. U. Mjörnmark, T. Moa, K. Mochizuki, S. Mohapatra, W. Mohr, S. Molander, R. Moles-Valls, R. Monden, K. Mönig, C. Monini, J. Monk, E. Monnier, A. Montalbano, J. Montejo Berlingen, F. Monticelli, S. Monzani, R. W. Moore, N. Morange, D. Moreno, M. Moreno Llácer, P. Morettini, D. Mori, T. Mori, M. Morii, M. Morinaga, V. Morisbak, S. Moritz, A. K. Morley, G. Mornacchi, J. D. Morris, S. S. Mortensen, A. Morton, L. Morvaj, M. Mosidze, J. Moss, K. Motohashi, R. Mount, E. Mountricha, S. V. Mouraviev, E. J. W. Moyse, S. Muanza, R. D. Mudd, F. Mueller, J. Mueller, R. S. P. Mueller, T. Mueller, D. Muenstermann, P. Mullen, G. A. Mullier, F. J. Munoz Sanchez, J. A. Murillo Quijada, W. J. Murray, H. Musheghyan, E. Musto, A. G. Myagkov, M. Myska, B. P. Nachman, O. Nackenhorst, J. Nadal, K. Nagai, R. Nagai, Y. Nagai, K. Nagano, A. Nagarkar, Y. Nagasaka, K. Nagata, M. Nagel, E. Nagy, A. M. Nairz, Y. Nakahama, K. Nakamura, T. Nakamura, I. Nakano, H. Namasivayam, R. F. Naranjo Garcia, R. Narayan, D. I. Narrias Villar, T. Naumann, G. Navarro, R. Nayyar, H. A. Neal, P. Yu. Nechaeva, T. J. Neep, P. D. Nef, A. Negri, M. Negrini, S. Nektarijevic, C. Nellist, A. Nelson, S. Nemecek, P. Nemethy, A. A. Nepomuceno, M. Nessi, M. S. Neubauer, M. Neumann, R. M. Neves, P. Nevski, P. R. Newman, D. H. Nguyen, R. B. Nickerson, R. Nicolaidou, B. Nicquevert, J. Nielsen, N. Nikiforou, A. Nikiforov, V. Nikolaenko, I. Nikolic-Audit, K. Nikolopoulos, J. K. Nilsen, P. Nilsson, Y. Ninomiya, A. Nisati, R. Nisius, T. Nobe, L. Nodulman, M. Nomachi, I. Nomidis, T. Nooney, S. Norberg, M. Nordberg, O. Novgorodova, S. Nowak, M. Nozaki, L. Nozka, K. Ntekas, G. Nunes Hanninger, T. Nunnemann, E. Nurse, F. Nuti, B. J. O’Brien, F. O’grady, D. C. O’Neil, V. O’Shea, F. G. Oakham, H. Oberlack, T. Obermann, J. Ocariz, A. Ochi, I. Ochoa, J. P. Ochoa-Ricoux, S. Oda, S. Odaka, H. Ogren, A. Oh, S. H. Oh, C. C. Ohm, H. Ohman, H. Oide, W. Okamura, H. Okawa, Y. Okumura, T. Okuyama, A. Olariu, S. A. Olivares Pino, D. Oliveira Damazio, A. Olszewski, J. Olszowska, A. Onofre, K. Onogi, P. U. E. Onyisi, C. J. Oram, M. J. Oreglia, Y. Oren, D. Orestano, N. Orlando, C. Oropeza Barrera, R. S. Orr, B. Osculati, R. Ospanov, G. Otero y Garzon, H. Otono, M. Ouchrif, F. Ould-Saada, A. Ouraou, K. P. Oussoren, Q. Ouyang, A. Ovcharova, M. Owen, R. E. Owen, V. E. Ozcan, N. Ozturk, K. Pachal, A. Pacheco Pages, C. Padilla Aranda, M. Pagáčová, S. Pagan Griso, E. Paganis, F. Paige, P. Pais, K. Pajchel, G. Palacino, S. Palestini, M. Palka, D. Pallin, A. Palma, Y. B. Pan, E. St. Panagiotopoulou, C. E. Pandini, J. G. Panduro Vazquez, P. Pani, S. Panitkin, D. Pantea, L. Paolozzi, Th. D. Papadopoulou, K. Papageorgiou, A. Paramonov, D. Paredes Hernandez, M. A. Parker, K. A. Parker, F. Parodi, J. A. Parsons, U. Parzefall, E. Pasqualucci, S. Passaggio, F. Pastore, Fr. Pastore, G. Pásztor, S. Pataraia, N. D. Patel, J. R. Pater, T. Pauly, J. Pearce, B. Pearson, L. E. Pedersen, M. Pedersen, S. Pedraza Lopez, R. Pedro, S. V. Peleganchuk, D. Pelikan, O. Penc, C. Peng, H. Peng, B. Penning, J. Penwell, D. V. Perepelitsa, E. Perez Codina, M. T. Pérez García-Estañ, L. Perini, H. Pernegger, S. Perrella, R. Peschke, V. D. Peshekhonov, K. Peters, R. F. Y. Peters, B. A. Petersen, T. C. Petersen, E. Petit, A. Petridis, C. Petridou, P. Petroff, E. Petrolo, F. Petrucci, N. E. Pettersson, R. Pezoa, P. W. Phillips, G. Piacquadio, E. Pianori, A. Picazio, E. Piccaro, M. Piccinini, M. A. Pickering, R. Piegaia, D. T. Pignotti, J. E. Pilcher, A. D. Pilkington, A. W. J. Pin, J. Pina, M. Pinamonti, J. L. Pinfold, A. Pingel, S. Pires, H. Pirumov, M. Pitt, C. Pizio, L. Plazak, M.-A. Pleier, V. Pleskot, E. Plotnikova, P. Plucinski, D. Pluth, R. Poettgen, L. Poggioli, D. Pohl, G. Polesello, A. Poley, A. Policicchio, R. Polifka, A. Polini, C. S. Pollard, V. Polychronakos, K. Pommès, L. Pontecorvo, B. G. Pope, G. A. Popeneciu, D. S. Popovic, A. Poppleton, S. Pospisil, K. Potamianos, I. N. Potrap, C. J. Potter, C. T. Potter, G. Poulard, J. Poveda, V. Pozdnyakov, P. Pralavorio, A. Pranko, S. Prasad, S. Prell, D. Price, L. E. Price, M. Primavera, S. Prince, M. Proissl, K. Prokofiev, F. Prokoshin, E. Protopapadaki, S. Protopopescu, J. Proudfoot, M. Przybycien, E. Ptacek, D. Puddu, E. Pueschel, D. Puldon, M. Purohit, P. Puzo, J. Qian, G. Qin, Y. Qin, A. Quadt, D. R. Quarrie, W. B. Quayle, M. Queitsch-Maitland, D. Quilty, S. Raddum, V. Radeka, V. Radescu, S. K. Radhakrishnan, P. Radloff, P. Rados, F. Ragusa, G. Rahal, S. Rajagopalan, M. Rammensee, C. Rangel-Smith, F. Rauscher, S. Rave, T. Ravenscroft, M. Raymond, A. L. Read, N. P. Readioff, D. M. Rebuzzi, A. Redelbach, G. Redlinger, R. Reece, K. Reeves, L. Rehnisch, J. Reichert, H. Reisin, C. Rembser, H. Ren, A. Renaud, M. Rescigno, S. Resconi, O. L. Rezanova, P. Reznicek, R. Rezvani, R. Richter, S. Richter, E. Richter-Was, O. Ricken, M. Ridel, P. Rieck, C. J. Riegel, J. Rieger, O. Rifki, M. Rijssenbeek, A. Rimoldi, L. Rinaldi, B. Ristić, E. Ritsch, I. Riu, F. Rizatdinova, E. Rizvi, S. H. Robertson, A. Robichaud-Veronneau, D. Robinson, J. E. M. Robinson, A. Robson, C. Roda, S. Roe, O. Røhne, A. Romaniouk, M. Romano, S. M. Romano Saez, E. Romero Adam, N. Rompotis, M. Ronzani, L. Roos, E. Ros, S. Rosati, K. Rosbach, P. Rose, P. L. Rosendahl, O. Rosenthal, V. Rossetti, E. Rossi, L. P. Rossi, J. H. N. Rosten, R. Rosten, M. Rotaru, I. Roth, J. Rothberg, D. Rousseau, C. R. Royon, A. Rozanov, Y. Rozen, X. Ruan, F. Rubbo, I. Rubinskiy, V. I. Rud, C. Rudolph, M. S. Rudolph, F. Rühr, A. Ruiz-Martinez, Z. Rurikova, N. A. Rusakovich, A. Ruschke, H. L. Russell, J. P. Rutherfoord, N. Ruthmann, Y. F. Ryabov, M. Rybar, G. Rybkin, N. C. Ryder, A. Ryzhov, A. F. Saavedra, G. Sabato, S. Sacerdoti, A. Saddique, H. F-W. Sadrozinski, R. Sadykov, F. Safai Tehrani, P. Saha, M. Sahinsoy, M. Saimpert, T. Saito, H. Sakamoto, Y. Sakurai, G. Salamanna, A. Salamon, J. E. Salazar Loyola, M. Saleem, D. Salek, P. H. Sales De Bruin, D. Salihagic, A. Salnikov, J. Salt, D. Salvatore, F. Salvatore, A. Salvucci, A. Salzburger, D. Sammel, D. Sampsonidis, A. Sanchez, J. Sánchez, V. Sanchez Martinez, H. Sandaker, R. L. Sandbach, H. G. Sander, M. P. Sanders, M. Sandhoff, C. Sandoval, R. Sandstroem, D. P. C. Sankey, M. Sannino, A. Sansoni, C. Santoni, R. Santonico, H. Santos, I. Santoyo Castillo, K. Sapp, A. Sapronov, J. G. Saraiva, B. Sarrazin, O. Sasaki, Y. Sasaki, K. Sato, G. Sauvage, E. Sauvan, G. Savage, P. Savard, C. Sawyer, L. Sawyer, J. Saxon, C. Sbarra, A. Sbrizzi, T. Scanlon, D. A. Scannicchio, M. Scarcella, V. Scarfone, J. Schaarschmidt, P. Schacht, D. Schaefer, R. Schaefer, J. Schaeffer, S. Schaepe, S. Schaetzel, U. Schäfer, A. C. Schaffer, D. Schaile, R. D. Schamberger, V. Scharf, V. A. Schegelsky, D. Scheirich, M. Schernau, C. Schiavi, C. Schillo, M. Schioppa, S. Schlenker, K. Schmieden, C. Schmitt, S. Schmitt, S. Schmitt, B. Schneider, Y. J. Schnellbach, U. Schnoor, L. Schoeffel, A. Schoening, B. D. Schoenrock, E. Schopf, A. L. S. Schorlemmer, M. Schott, D. Schouten, J. Schovancova, S. Schramm, M. Schreyer, N. Schuh, M. J. Schultens, H.-C. Schultz-Coulon, H. Schulz, M. Schumacher, B. A. Schumm, Ph. Schune, C. Schwanenberger, A. Schwartzman, T. A. Schwarz, Ph. Schwegler, H. Schweiger, Ph. Schwemling, R. Schwienhorst, J. Schwindling, T. Schwindt, F. G. Sciacca, E. Scifo, G. Sciolla, F. Scuri, F. Scutti, J. Searcy, G. Sedov, E. Sedykh, P. Seema, S. C. Seidel, A. Seiden, F. Seifert, J. M. Seixas, G. Sekhniaidze, K. Sekhon, S. J. Sekula, D. M. Seliverstov, N. Semprini-Cesari, C. Serfon, L. Serin, L. Serkin, T. Serre, M. Sessa, R. Seuster, H. Severini, T. Sfiligoj, F. Sforza, A. Sfyrla, E. Shabalina, M. Shamim, L. Y. Shan, R. Shang, J. T. Shank, M. Shapiro, P. B. Shatalov, K. Shaw, S. M. Shaw, A. Shcherbakova, C. Y. Shehu, P. Sherwood, L. Shi, S. Shimizu, C. O. Shimmin, M. Shimojima, M. Shiyakova, A. Shmeleva, D. Shoaleh Saadi, M. J. Shochet, S. Shojaii, S. Shrestha, E. Shulga, M. A. Shupe, S. Shushkevich, P. Sicho, P. E. Sidebo, O. Sidiropoulou, D. Sidorov, A. Sidoti, F. Siegert, Dj. Sijacki, J. Silva, Y. Silver, S. B. Silverstein, V. Simak, O. Simard, Lj. Simic, S. Simion, E. Simioni, B. Simmons, D. Simon, P. Sinervo, N. B. Sinev, M. Sioli, G. Siragusa, A. N. Sisakyan, S. Yu. Sivoklokov, J. Sjölin, T. B. Sjursen, M. B. Skinner, H. P. Skottowe, P. Skubic, M. Slater, T. Slavicek, M. Slawinska, K. Sliwa, V. Smakhtin, B. H. Smart, L. Smestad, S. Yu. Smirnov, Y. Smirnov, L. N. Smirnova, O. Smirnova, M. N. K. Smith, R. W. Smith, M. Smizanska, K. Smolek, A. A. Snesarev, G. Snidero, S. Snyder, R. Sobie, F. Socher, A. Soffer, D. A. Soh, G. Sokhrannyi, C. A. Solans Sanchez, M. Solar, J. Solc, E. Yu. Soldatov, U. Soldevila, A. A. Solodkov, A. Soloshenko, O. V. Solovyanov, V. Solovyev, P. Sommer, H. Y. Song, N. Soni, A. Sood, A. Sopczak, B. Sopko, V. Sopko, V. Sorin, D. Sosa, M. Sosebee, C. L. Sotiropoulou, R. Soualah, A. M. Soukharev, D. South, B. C. Sowden, S. Spagnolo, M. Spalla, M. Spangenberg, F. Spanò, W. R. Spearman, D. Sperlich, F. Spettel, R. Spighi, G. Spigo, L. A. Spiller, M. Spousta, R. D. St. Denis, A. Stabile, S. Staerz, J. Stahlman, R. Stamen, S. Stamm, E. Stanecka, R. W. Stanek, C. Stanescu, M. Stanescu-Bellu, M. M. Stanitzki, S. Stapnes, E. A. Starchenko, J. Stark, P. Staroba, P. Starovoitov, R. Staszewski, P. Steinberg, B. Stelzer, H. J. Stelzer, O. Stelzer-Chilton, H. Stenzel, G. A. Stewart, J. A. Stillings, M. C. Stockton, M. Stoebe, G. Stoicea, P. Stolte, S. Stonjek, A. R. Stradling, A. Straessner, M. E. Stramaglia, J. Strandberg, S. Strandberg, A. Strandlie, E. Strauss, M. Strauss, P. Strizenec, R. Ströhmer, D. M. Strom, R. Stroynowski, A. Strubig, S. A. Stucci, B. Stugu, N. A. Styles, D. Su, J. Su, R. Subramaniam, A. Succurro, S. Suchek, Y. Sugaya, M. Suk, V. V. Sulin, S. Sultansoy, T. Sumida, S. Sun, X. Sun, J. E. Sundermann, K. Suruliz, G. Susinno, M. R. Sutton, S. Suzuki, M. Svatos, M. Swiatlowski, I. Sykora, T. Sykora, D. Ta, C. Taccini, K. Tackmann, J. Taenzer, A. Taffard, R. Tafirout, N. Taiblum, H. Takai, R. Takashima, H. Takeda, T. Takeshita, Y. Takubo, M. Talby, A. A. Talyshev, J. Y. C. Tam, K. G. Tan, J. Tanaka, R. Tanaka, S. Tanaka, B. B. Tannenwald, N. Tannoury, S. Tapia Araya, S. Tapprogge, S. Tarem, F. Tarrade, G. F. Tartarelli, P. Tas, M. Tasevsky, T. Tashiro, E. Tassi, A. Tavares Delgado, Y. Tayalati, F. E. Taylor, G. N. Taylor, P. T. E. Taylor, W. Taylor, F. A. Teischinger, P. Teixeira-Dias, K. K. Temming, D. Temple, H. Ten Kate, P. K. Teng, J. J. Teoh, F. Tepel, S. Terada, K. Terashi, J. Terron, S. Terzo, M. Testa, R. J. Teuscher, T. Theveneaux-Pelzer, J. P. Thomas, J. Thomas-Wilsker, E. N. Thompson, P. D. Thompson, R. J. Thompson, A. S. Thompson, L. A. Thomsen, E. Thomson, M. Thomson, R. P. Thun, M. J. Tibbetts, R. E. Ticse Torres, V. O. Tikhomirov, Yu. A. Tikhonov, S. Timoshenko, E. Tiouchichine, P. Tipton, S. Tisserant, K. Todome, T. Todorov, S. Todorova-Nova, J. Tojo, S. Tokár, K. Tokushuku, K. Tollefson, E. Tolley, L. Tomlinson, M. Tomoto, L. Tompkins, K. Toms, E. Torrence, H. Torres, E. Torró Pastor, J. Toth, F. Touchard, D. R. Tovey, T. Trefzger, L. Tremblet, A. Tricoli, I. M. Trigger, S. Trincaz-Duvoid, M. F. Tripiana, W. Trischuk, B. Trocmé, C. Troncon, M. Trottier-McDonald, M. Trovatelli, L. Truong, M. Trzebinski, A. Trzupek, C. Tsarouchas, J. C-L. Tseng, P. V. Tsiareshka, D. Tsionou, G. Tsipolitis, N. Tsirintanis, S. Tsiskaridze, V. Tsiskaridze, E. G. Tskhadadze, K. M. Tsui, I. I. Tsukerman, V. Tsulaia, S. Tsuno, D. Tsybychev, A. Tudorache, V. Tudorache, A. N. Tuna, S. A. Tupputi, S. Turchikhin, D. Turecek, R. Turra, A. J. Turvey, P. M. Tuts, A. Tykhonov, M. Tylmad, M. Tyndel, I. Ueda, R. Ueno, M. Ughetto, M. Ugland, F. Ukegawa, G. Unal, A. Undrus, G. Unel, F. C. Ungaro, Y. Unno, C. Unverdorben, J. Urban, P. Urquijo, P. Urrejola, G. Usai, A. Usanova, L. Vacavant, V. Vacek, B. Vachon, C. Valderanis, N. Valencic, S. Valentinetti, A. Valero, L. Valery, S. Valkar, S. Vallecorsa, J. A. Valls Ferrer, W. Van Den Wollenberg, P. C. Van Der Deijl, R. van der Geer, H. van der Graaf, N. van Eldik, P. van Gemmeren, J. Van Nieuwkoop, I. van Vulpen, M. C. van Woerden, M. Vanadia, W. Vandelli, R. Vanguri, A. Vaniachine, F. Vannucci, G. Vardanyan, R. Vari, E. W. Varnes, T. Varol, D. Varouchas, A. Vartapetian, K. E. Varvell, F. Vazeille, T. Vazquez Schroeder, J. Veatch, L. M. Veloce, F. Veloso, T. Velz, S. Veneziano, A. Ventura, D. Ventura, M. Venturi, N. Venturi, A. Venturini, V. Vercesi, M. Verducci, W. Verkerke, J. C. Vermeulen, A. Vest, M. C. Vetterli, O. Viazlo, I. Vichou, T. Vickey, O. E. Vickey Boeriu, G. H. A. Viehhauser, S. Viel, R. Vigne, M. Villa, M. Villaplana Perez, E. Vilucchi, M. G. Vincter, V. B. Vinogradov, I. Vivarelli, F. Vives Vaque, S. Vlachos, D. Vladoiu, M. Vlasak, M. Vogel, P. Vokac, G. Volpi, M. Volpi, H. von der Schmitt, H. von Radziewski, E. von Toerne, V. Vorobel, K. Vorobev, M. Vos, R. Voss, J. H. Vossebeld, N. Vranjes, M. Vranjes Milosavljevic, V. Vrba, M. Vreeswijk, R. Vuillermet, I. Vukotic, Z. Vykydal, P. Wagner, W. Wagner, H. Wahlberg, S. Wahrmund, J. Wakabayashi, J. Walder, R. Walker, W. Walkowiak, C. Wang, F. Wang, H. Wang, H. Wang, J. Wang, J. Wang, K. Wang, R. Wang, S. M. Wang, T. Wang, T. Wang, X. Wang, C. Wanotayaroj, A. Warburton, C. P. Ward, D. R. Wardrope, A. Washbrook, C. Wasicki, P. M. Watkins, A. T. Watson, I. J. Watson, M. F. Watson, G. Watts, S. Watts, B. M. Waugh, S. Webb, M. S. Weber, S. W. Weber, J. S. Webster, A. R. Weidberg, B. Weinert, J. Weingarten, C. Weiser, H. Weits, P. S. Wells, T. Wenaus, T. Wengler, S. Wenig, N. Wermes, M. Werner, P. Werner, M. Wessels, J. Wetter, K. Whalen, A. M. Wharton, A. White, M. J. White, R. White, S. White, D. Whiteson, F. J. Wickens, W. Wiedenmann, M. Wielers, P. Wienemann, C. Wiglesworth, L. A. M. Wiik-Fuchs, A. Wildauer, H. G. Wilkens, H. H. Williams, S. Williams, C. Willis, S. Willocq, A. Wilson, J. A. Wilson, I. Wingerter-Seez, F. Winklmeier, B. T. Winter, M. Wittgen, J. Wittkowski, S. J. Wollstadt, M. W. Wolter, H. Wolters, B. K. Wosiek, J. Wotschack, M. J. Woudstra, K. W. Wozniak, M. Wu, M. Wu, S. L. Wu, X. Wu, Y. Wu, T. R. Wyatt, B. M. Wynne, S. Xella, D. Xu, L. Xu, B. Yabsley, S. Yacoob, R. Yakabe, M. Yamada, D. Yamaguchi, Y. Yamaguchi, A. Yamamoto, S. Yamamoto, T. Yamanaka, K. Yamauchi, Y. Yamazaki, Z. Yan, H. Yang, H. Yang, Y. Yang, W-M. Yao, Y. C. Yap, Y. Yasu, E. Yatsenko, K. H. Yau Wong, J. Ye, S. Ye, I. Yeletskikh, A. L. Yen, E. Yildirim, K. Yorita, R. Yoshida, K. Yoshihara, C. Young, C. J. S. Young, S. Youssef, D. R. Yu, J. Yu, J. M. Yu, J. Yu, L. Yuan, S. P. Y. Yuen, A. Yurkewicz, I. Yusuff, B. Zabinski, R. Zaidan, A. M. Zaitsev, J. Zalieckas, A. Zaman, S. Zambito, L. Zanello, D. Zanzi, C. Zeitnitz, M. Zeman, A. Zemla, Q. Zeng, K. Zengel, O. Zenin, T. Ženiš, D. Zerwas, D. Zhang, F. Zhang, G. Zhang, H. Zhang, J. Zhang, L. Zhang, R. Zhang, X. Zhang, Z. Zhang, X. Zhao, Y. Zhao, Z. Zhao, A. Zhemchugov, J. Zhong, B. Zhou, C. Zhou, L. Zhou, L. Zhou, M. Zhou, N. Zhou, C. G. Zhu, H. Zhu, J. Zhu, Y. Zhu, X. Zhuang, K. Zhukov, A. Zibell, D. Zieminska, N. I. Zimine, C. Zimmermann, S. Zimmermann, Z. Zinonos, M. Zinser, M. Ziolkowski, L. Živković, G. Zobernig, A. Zoccoli, M. zur Nedden, G. Zurzolo, L. Zwalinski

**Affiliations:** 1 Department of Physics, University of Adelaide, Adelaide, Australia; 2 Physics Department, SUNY Albany, Albany, NY USA; 3 Department of Physics, University of Alberta, Edmonton, AB Canada; 4 Department of Physics, Ankara University, Ankara, Turkey; 5 Istanbul Aydin University, Istanbul, Turkey; 6 Division of Physics, TOBB University of Economics and Technology, Ankara, Turkey; 7 LAPP, CNRS/IN2P3 and Université Savoie Mont Blanc, Annecy-le-Vieux, France; 8 High Energy Physics Division, Argonne National Laboratory, Argonne, IL USA; 9 Department of Physics, University of Arizona, Tucson, AZ USA; 10 Department of Physics, The University of Texas at Arlington, Arlington, TX USA; 11 Physics Department, University of Athens, Athens, Greece; 12 Physics Department, National Technical University of Athens, Zografou, Greece; 13 Institute of Physics, Azerbaijan Academy of Sciences, Baku, Azerbaijan; 14 Institut de Física d’Altes Energies (IFAE), The Barcelona Institute of Science and Technology, Barcelona, Spain; 15 Institute of Physics, University of Belgrade, Belgrade, Serbia; 16 Department for Physics and Technology, University of Bergen, Bergen, Norway; 17 Physics Division, Lawrence Berkeley National Laboratory and University of California, Berkeley, CA USA; 18 Department of Physics, Humboldt University, Berlin, Germany; 19 Albert Einstein Center for Fundamental Physics and Laboratory for High Energy Physics, University of Bern, Bern, Switzerland; 20 School of Physics and Astronomy, University of Birmingham, Birmingham, UK; 21 Department of Physics, Bogazici University, Istanbul, Turkey; 22 Department of Physics Engineering, Gaziantep University, Gaziantep, Turkey; 23 Department of Physics, Dogus University, Istanbul, Turkey; 24 Centro de Investigaciones, Universidad Antonio Narino, Bogota, Colombia; 25 INFN Sezione di Bologna, Bologna, Italy; 26 Dipartimento di Fisica e Astronomia, Università di Bologna, Bologna, Italy; 27 Physikalisches Institut, University of Bonn, Bonn, Germany; 28 Department of Physics, Boston University, Boston, MA USA; 29 Department of Physics, Brandeis University, Waltham, MA USA; 30 Universidade Federal do Rio De Janeiro COPPE/EE/IF, Rio de Janeiro, Brazil; 31 Electrical Circuits Department, Federal University of Juiz de Fora (UFJF), Juiz de Fora, Brazil; 32 Federal University of Sao Joao del Rei (UFSJ), Sao Joao del Rei, Brazil; 33 Instituto de Fisica, Universidade de Sao Paulo, São Paulo, Brazil; 34 Physics Department, Brookhaven National Laboratory, Upton, NY USA; 35 Transilvania University of Brasov, National Institute of Physics and Nuclear Engineering, Brasov, Romania; 36 National Institute of Physics and Nuclear Engineering, Bucharest, Romania; 37 Physics Department, National Institute for Research and Development of Isotopic and Molecular Technologies, Cluj Napoca, Romania; 38 University Politehnica Bucharest, Bucharest, Romania; 39 West University in Timisoara, Timisoara, Romania; 40 Departamento de Física, Universidad de Buenos Aires, Buenos Aires, Argentina; 41 Cavendish Laboratory, University of Cambridge, Cambridge, UK; 42 Department of Physics, Carleton University, Ottawa, ON Canada; 43 CERN, Geneva, Switzerland; 44 Enrico Fermi Institute, University of Chicago, Chicago, IL USA; 45 Departamento de Física, Pontificia Universidad Católica de Chile, Santiago, Chile; 46 Departamento de Física, Universidad Técnica Federico Santa María, Valparaiso, Chile; 47 Institute of High Energy Physics, Chinese Academy of Sciences, Beijing, China; 48 Department of Modern Physics, University of Science and Technology of China, Hefei, Anhui China; 49 Department of Physics, Nanjing University, Nanjing, Jiangsu China; 50 School of Physics, Shandong University, Jinan, Shandong China; 51 Shanghai Key Laboratory for Particle Physics and Cosmology, Department of Physics and Astronomy, Shanghai Jiao Tong University (also affiliated with PKU-CHEP), Shanghai, China; 52 Physics Department, Tsinghua University, Beijing, 100084 China; 53 Laboratoire de Physique Corpusculaire, Clermont Université and Université Blaise Pascal and CNRS/IN2P3, Clermont-Ferrand, France; 54 Nevis Laboratory, Columbia University, Irvington, NY USA; 55 Niels Bohr Institute, University of Copenhagen, Copenhagen, Denmark; 56 INFN Gruppo Collegato di Cosenza, Laboratori Nazionali di Frascati, Frascati, Italy; 57 Dipartimento di Fisica, Università della Calabria, Rende, Italy; 58 Faculty of Physics and Applied Computer Science, AGH University of Science and Technology, Kraków, Poland; 59 Marian Smoluchowski Institute of Physics, Jagiellonian University, Kraków, Poland; 60 Institute of Nuclear Physics, Polish Academy of Sciences, Kraków, Poland; 61 Physics Department, Southern Methodist University, Dallas, TX USA; 62 Physics Department, University of Texas at Dallas, Richardson, TX USA; 63 DESY, Hamburg and Zeuthen, Germany; 64 Institut für Experimentelle Physik IV, Technische Universität Dortmund, Dortmund, Germany; 65 Institut für Kern- und Teilchenphysik, Technische Universität Dresden, Dresden, Germany; 66 Department of Physics, Duke University, Durham, NC USA; 67 SUPA-School of Physics and Astronomy, University of Edinburgh, Edinburgh, UK; 68 INFN Laboratori Nazionali di Frascati, Frascati, Italy; 69 Fakultät für Mathematik und Physik, Albert-Ludwigs-Universität, Freiburg, Germany; 70 Section de Physique, Université de Genève, Geneva, Switzerland; 71 INFN Sezione di Genova, Genoa, Italy; 72 Dipartimento di Fisica, Università di Genova, Genoa, Italy; 73 E. Andronikashvili Institute of Physics, Iv. Javakhishvili Tbilisi State University, Tbilisi, Georgia; 74 High Energy Physics Institute, Tbilisi State University, Tbilisi, Georgia; 75 II Physikalisches Institut, Justus-Liebig-Universität Giessen, Giessen, Germany; 76 SUPA-School of Physics and Astronomy, University of Glasgow, Glasgow, UK; 77 II Physikalisches Institut, Georg-August-Universität, Göttingen, Germany; 78 Laboratoire de Physique Subatomique et de Cosmologie, Université Grenoble-Alpes, CNRS/IN2P3, Grenoble, France; 79 Department of Physics, Hampton University, Hampton, VA USA; 80 Laboratory for Particle Physics and Cosmology, Harvard University, Cambridge, MA USA; 81 Kirchhoff-Institut für Physik, Ruprecht-Karls-Universität Heidelberg, Heidelberg, Germany; 82 Physikalisches Institut, Ruprecht-Karls-Universität Heidelberg, Heidelberg, Germany; 83 ZITI Institut für technische Informatik, Ruprecht-Karls-Universität Heidelberg, Mannheim, Germany; 84 Faculty of Applied Information Science, Hiroshima Institute of Technology, Hiroshima, Japan; 85 Department of Physics, The Chinese University of Hong Kong, Shatin, NT Hong Kong; 86 Department of Physics, The University of Hong Kong, Pokfulam, Hong Kong; 87 Department of Physics, The Hong Kong University of Science and Technology, Clear Water Bay, Kowloon, Hong Kong China; 88 Department of Physics, Indiana University, Bloomington, IN USA; 89 Institut für Astro- und Teilchenphysik, Leopold-Franzens-Universität, Innsbruck, Austria; 90 University of Iowa, Iowa City, IA USA; 91 Department of Physics and Astronomy, Iowa State University, Ames, IA USA; 92 Joint Institute for Nuclear Research, JINR Dubna, Dubna, Russia; 93 KEK, High Energy Accelerator Research Organization, Tsukuba, Japan; 94 Graduate School of Science, Kobe University, Kobe, Japan; 95 Faculty of Science, Kyoto University, Kyoto, Japan; 96 Kyoto University of Education, Kyoto, Japan; 97 Department of Physics, Kyushu University, Fukuoka, Japan; 98 Instituto de Física La Plata, Universidad Nacional de La Plata and CONICET, La Plata, Argentina; 99 Physics Department, Lancaster University, Lancaster, UK; 100 INFN Sezione di Lecce, Lecce, Italy; 101 Dipartimento di Matematica e Fisica, Università del Salento, Lecce, Italy; 102 Oliver Lodge Laboratory, University of Liverpool, Liverpool, UK; 103 Department of Physics, Jožef Stefan Institute and University of Ljubljana, Ljubljana, Slovenia; 104 School of Physics and Astronomy, Queen Mary University of London, London, UK; 105 Department of Physics, Royal Holloway University of London, Surrey, UK; 106 Department of Physics and Astronomy, University College London, London, UK; 107 Louisiana Tech University, Ruston, LA USA; 108 Laboratoire de Physique Nucléaire et de Hautes Energies, UPMC and Université Paris-Diderot and CNRS/IN2P3, Paris, France; 109 Fysiska institutionen, Lunds universitet, Lund, Sweden; 110 Departamento de Fisica Teorica C-15, Universidad Autonoma de Madrid, Madrid, Spain; 111 Institut für Physik, Universität Mainz, Mainz, Germany; 112 School of Physics and Astronomy, University of Manchester, Manchester, UK; 113 CPPM, Aix-Marseille Université and CNRS/IN2P3, Marseille, France; 114 Department of Physics, University of Massachusetts, Amherst, MA USA; 115 Department of Physics, McGill University, Montreal, QC Canada; 116 School of Physics, University of Melbourne, Melbourne, VIC Australia; 117 Department of Physics, The University of Michigan, Ann Arbor, MI USA; 118 Department of Physics and Astronomy, Michigan State University, East Lansing, MI USA; 119 INFN Sezione di Milano, Milan, Italy; 120 Dipartimento di Fisica, Università di Milano, Milan, Italy; 121 B.I. Stepanov Institute of Physics, National Academy of Sciences of Belarus, Minsk, Republic of Belarus; 122 National Scientific and Educational Centre for Particle and High Energy Physics, Minsk, Republic of Belarus; 123 Department of Physics, Massachusetts Institute of Technology, Cambridge, MA USA; 124 Group of Particle Physics, University of Montreal, Montreal, QC Canada; 125 P.N. Lebedev Physical Institute of the Russian, Academy of Sciences, Moscow, Russia; 126 Institute for Theoretical and Experimental Physics (ITEP), Moscow, Russia; 127 National Research Nuclear University MEPhI, Moscow, Russia; 128 D.V. Skobeltsyn Institute of Nuclear Physics, M.V. Lomonosov Moscow State University, Moscow, Russia; 129 Fakultät für Physik, Ludwig-Maximilians-Universität München, Munich, Germany; 130 Max-Planck-Institut für Physik (Werner-Heisenberg-Institut), Munich, Germany; 131 Nagasaki Institute of Applied Science, Nagasaki, Japan; 132 Graduate School of Science and Kobayashi-Maskawa Institute, Nagoya University, Nagoya, Japan; 133 INFN Sezione di Napoli, Naples, Italy; 134 Dipartimento di Fisica, Università di Napoli, Naples, Italy; 135 Department of Physics and Astronomy, University of New Mexico, Albuquerque, NM USA; 136 Institute for Mathematics, Astrophysics and Particle Physics, Radboud University Nijmegen/Nikhef, Nijmegen, The Netherlands; 137 Nikhef National Institute for Subatomic Physics and University of Amsterdam, Amsterdam, The Netherlands; 138 Department of Physics, Northern Illinois University, De Kalb, IL USA; 139 Budker Institute of Nuclear Physics, SB RAS, Novosibirsk, Russia; 140 Department of Physics, New York University, New York, NY USA; 141 Ohio State University, Columbus, OH USA; 142 Faculty of Science, Okayama University, Okayama, Japan; 143 Homer L. Dodge Department of Physics and Astronomy, University of Oklahoma, Norman, OK USA; 144 Department of Physics, Oklahoma State University, Stillwater, OK USA; 145 Palacký University, RCPTM, Olomouc, Czech Republic; 146 Center for High Energy Physics, University of Oregon, Eugene, OR USA; 147 LAL, Univ. Paris-Sud, CNRS/IN2P3 Université Paris-Saclay, Orsay, France; 148 Graduate School of Science, Osaka University, Osaka, Japan; 149 Department of Physics, University of Oslo, Oslo, Norway; 150 Department of Physics, Oxford University, Oxford, UK; 151 INFN Sezione di Pavia, Pavia, Italy; 152 Dipartimento di Fisica, Università di Pavia, Pavia, Italy; 153 Department of Physics, University of Pennsylvania, Philadelphia, PA USA; 154 National Research Centre “Kurchatov Institute” B.P.Konstantinov Petersburg Nuclear Physics Institute, St. Petersburg, Russia; 155 INFN Sezione di Pisa, Pisa, Italy; 156 Dipartimento di Fisica E. Fermi, Università di Pisa, Pisa, Italy; 157 Department of Physics and Astronomy, University of Pittsburgh, Pittsburgh, PA USA; 158 Laboratório de Instrumentação e Física Experimental de Partículas-LIP, Lisbon, Portugal; 159 Faculdade de Ciências, Universidade de Lisboa, Lisbon, Portugal; 160 Department of Physics, University of Coimbra, Coimbra, Portugal; 161 Centro de Física Nuclear da Universidade de Lisboa, Lisbon, Portugal; 162 Departamento de Fisica, Universidade do Minho, Braga, Portugal; 163 Departamento de Fisica Teorica y del Cosmos and CAFPE, Universidad de Granada, Granada, Spain; 164 Dep Fisica and CEFITEC of Faculdade de Ciencias e Tecnologia, Universidade Nova de Lisboa, Caparica, Portugal; 165 Institute of Physics, Academy of Sciences of the Czech Republic, Prague, Czech Republic; 166 Czech Technical University in Prague, Prague, Czech Republic; 167 Faculty of Mathematics and Physics, Charles University in Prague, Prague, Czech Republic; 168 State Research Center Institute for High Energy Physics (Protvino), NRC KI, Protvino, Russia Russia; 169 Particle Physics Department, Rutherford Appleton Laboratory, Didcot, UK; 170 INFN Sezione di Roma, Rome, Italy; 171 Dipartimento di Fisica, Sapienza Università di Roma, Rome, Italy; 172 INFN Sezione di Roma Tor Vergata, Rome, Italy; 173 Dipartimento di Fisica, Università di Roma Tor Vergata, Rome, Italy; 174 INFN Sezione di Roma Tre, Rome, Italy; 175 Dipartimento di Matematica e Fisica, Università Roma Tre, Rome, Italy; 176 Faculté des Sciences Ain Chock, Réseau Universitaire de Physique des Hautes Energies-Université Hassan II, Casablanca, Morocco; 177 Centre National de l’Energie des Sciences Techniques Nucleaires, Rabat, Morocco; 178 Faculté des Sciences Semlalia, Université Cadi Ayyad, LPHEA-Marrakech, Marrakech, Morocco; 179 Faculté des Sciences, Université Mohamed Premier and LPTPM, Oujda, Morocco; 180 Faculté des Sciences, Université Mohammed V, Rabat, Morocco; 181 DSM/IRFU (Institut de Recherches sur les Lois Fondamentales de l’Univers), CEA Saclay (Commissariat à l’Energie Atomique et aux Energies Alternatives), Gif-sur-Yvette, France; 182 Santa Cruz Institute for Particle Physics, University of California Santa Cruz, Santa Cruz, CA USA; 183 Department of Physics, University of Washington, Seattle, WA USA; 184 Department of Physics and Astronomy, University of Sheffield, Sheffield, UK; 185 Department of Physics, Shinshu University, Nagano, Japan; 186 Fachbereich Physik, Universität Siegen, Siegen, Germany; 187 Department of Physics, Simon Fraser University, Burnaby, BC Canada; 188 SLAC National Accelerator Laboratory, Stanford, CA USA; 189 Faculty of Mathematics, Physics and Informatics, Comenius University, Bratislava, Slovak Republic; 190 Department of Subnuclear Physics, Institute of Experimental Physics of the Slovak Academy of Sciences, Kosice, Slovak Republic; 191 Department of Physics, University of Cape Town, Cape Town, South Africa; 192 Department of Physics, University of Johannesburg, Johannesburg, South Africa; 193 School of Physics, University of the Witwatersrand, Johannesburg, South Africa; 194 Department of Physics, Stockholm University, Stockholm, Sweden; 195 The Oskar Klein Centre, Stockholm, Sweden; 196 Physics Department, Royal Institute of Technology, Stockholm, Sweden; 197 Departments of Physics and Astronomy and Chemistry, Stony Brook University, Stony Brook, NY USA; 198 Department of Physics and Astronomy, University of Sussex, Brighton, UK; 199 School of Physics, University of Sydney, Sydney, Australia; 200 Institute of Physics, Academia Sinica, Taipei, Taiwan; 201 Department of Physics, Technion: Israel Institute of Technology, Haifa, Israel; 202 Raymond and Beverly Sackler School of Physics and Astronomy, Tel Aviv University, Tel Aviv, Israel; 203 Department of Physics, Aristotle University of Thessaloniki, Thessaloníki, Greece; 204 International Center for Elementary Particle Physics and Department of Physics, The University of Tokyo, Tokyo, Japan; 205 Graduate School of Science and Technology, Tokyo Metropolitan University, Tokyo, Japan; 206 Department of Physics, Tokyo Institute of Technology, Tokyo, Japan; 207 Department of Physics, University of Toronto, Toronto, ON Canada; 208 TRIUMF, Vancouver, BC Canada; 209 Department of Physics and Astronomy, York University, Toronto, ON Canada; 210 Faculty of Pure and Applied Sciences and Center for Integrated Research in Fundamental Science and Engineering, University of Tsukuba, Tsukuba, Japan; 211 Department of Physics and Astronomy, Tufts University, Medford, MA USA; 212 Department of Physics and Astronomy, University of California Irvine, Irvine, CA USA; 213 INFN Gruppo Collegato di Udine, Sezione di Trieste, Udine, Italy; 214 ICTP, Trieste, Italy; 215 Dipartimento di Chimica Fisica e Ambiente, Università di Udine, Udine, Italy; 216 Department of Physics and Astronomy, University of Uppsala, Uppsala, Sweden; 217 Department of Physics, University of Illinois, Urbana, IL USA; 218 Instituto de Física Corpuscular (IFIC) and Departamento de Física Atómica, Molecular y Nuclear and Departamento de Ingeniería Electrónica and Instituto de Microelectrónica de Barcelona (IMB-CNM), University of Valencia and CSIC, Valencia, Spain; 219 Department of Physics, University of British Columbia, Vancouver, BC Canada; 220 Department of Physics and Astronomy, University of Victoria, Victoria, BC Canada; 221 Department of Physics, University of Warwick, Coventry, UK; 222 Waseda University, Tokyo, Japan; 223 Department of Particle Physics, The Weizmann Institute of Science, Rehovot, Israel; 224 Department of Physics, University of Wisconsin, Madison, WI USA; 225 Fakultät für Physik und Astronomie, Julius-Maximilians-Universität, Würzburg, Germany; 226 Fachbereich C Fakultät für Mathematik und Naturwissenschaften, Fachgruppe Physik, Bergische Universität Wuppertal, Wuppertal, Germany; 227 Department of Physics, Yale University, New Haven, CT USA; 228 Yerevan Physics Institute, Yerevan, Armenia; 229 Centre de Calcul de l’Institut National de Physique Nucléaire et de Physique des Particules (IN2P3), Villeurbanne, France; 230 CERN, Geneva, Switzerland

## Abstract

This article presents the sensitivity of the ATLAS experiment to the lepton-flavour-violating decays of $\tau \rightarrow 3\mu$. A method utilising the production of $\tau$ leptons via $W\rightarrow \tau \nu$ decays is used. This method is applied to the sample of 20.3 fb$^{-1}$ of *pp* collision data at a centre-of-mass energy of 8 TeV collected by the ATLAS experiment at the LHC in 2012. No event is observed passing the selection criteria, and the observed (expected) upper limit on the $\tau$ lepton branching fraction into three muons, $\mathrm{Br}(\tau \rightarrow 3\mu )$, is $3.76\times 10^{-7}$ ($3.94\times 10^{-7}$) at 90 % confidence level.

## Introduction

The observation of a lepton-flavour-violating (LFV) process involving charged leptons would be a major breakthrough in understanding the matter content of the universe and would support the hypothesis of leptogenesis [1]. In particular, LFV processes involving both a $\tau$ lepton and a muon are seen as most promising for such an observation, given the current measurements of neutrino oscillations [2]. In the Standard Model (SM), such processes have a vanishingly small branching fraction, e.g. $\mathrm{Br}(\tau \rightarrow 3\mu )$ $<10^{-14}$ [3], while a number of models beyond the SM predict it to be of the order of $10^{-10}$–$10^{-8}$ [4–6]. The current limits on branching fractions of neutrinoless $\tau$ lepton decays are of the order of few times $10^{-8}$ [7–10], for *Z* boson LFV decays they are about $10^{-5}$ [2, 11, 12], and for the LFV decay of a Higgs boson to a $\tau$ lepton and a muon they are about 1 % [13, 14]. The main experimental obstacles to improve the sensitivity with $\tau$ leptons are the small number of produced $\tau$ leptons world-wide.

In this article, a search for neutrinoless $\tau$ lepton decays to three muons is performed with 20.3 fb$^{-1}$ of *pp* collision data collected with ATLAS detector in 2012 at 8 TeV centre-of-mass energy. The search is focused on a particular source of $\tau$ leptons, namely $W\rightarrow \tau \nu$ decays with subsequent $\tau \rightarrow 3\mu$ decay. In such events, $\tau$ leptons are produced with a transverse momentum ($p_\mathrm{T}$) mostly in the range of ${\sim }25{-}50$ GeV. Due to the relativistic boost of the $\tau$ lepton, the muons from the $\tau$ LFV decay are produced in close geometrical proximity to each other but isolated from other energetic particles in the event. The tau-neutrino from the *W* boson decay appears as missing transverse momentum ($E_\mathrm{T}^\mathrm{miss}$) in the detector and together with the transverse momentum of the three muons ($p_\mathrm{T}^{3\mu }$) gives a transverse mass, $m_\mathrm{T}=\sqrt{2p_\mathrm{T}^{3\mu }E_\mathrm{T}^\mathrm{miss} (1-\cos \Delta \phi )}$, compatible with the *W* boson decay, where $\Delta \phi$ is the angle between the directions of the $p_\mathrm{T}^{3\mu }$ and the $E_\mathrm{T}^\mathrm{miss}$. The unique signature in the detector is three muons with invariant mass equal to the mass of the $\tau$ lepton and with a significant missing transverse momentum that is on average back-to-back with the three muons in the transverse plane. Since no energetic jet is expected in the majority of *W* boson production events, very small hadronic activity is predicted beyond that from the soft underlying event or multiple simultaneous *pp* collisions (pile-up). A large fraction of such $\tau$ leptons decay sufficiently far from the *W* production vertex to give a fully reconstructable additional vertex. This allows the selection of three muons originating from a vertex which is displaced from the primary interaction vertex. The background events usually contain one or two muons originating from the decay of hadrons, including decays in flight, while the remaining tracks are hadrons mis-measured as muons, originating from e.g. a pile-up jet or a pion punching through the calorimeter. The dominant background is due to muons originating from decays of *b*- or *c*-hadrons (heavy flavour, HF). Although such decays are typically accompanied by jets of particles produced in the direction opposite to the HF jets, in a fraction of the events the associated jet is lost or mis-measured, mimicking the signal $E_\mathrm{T}^\mathrm{miss}$. A small light-flavour multi-jet contribution is also present while the contribution from leptonic decays of vector bosons is negligible.

The analysis strategy is as follows. Events with three muons associated with a common vertex are selected. A loose event selection is applied to collect a high-quality sample of candidate events satisfying $|m_{3\mu }-m_\tau |\lesssim 1$ GeV. The characteristics of the loose sample of events are then analysed with a *boosted decision tree* (BDT). The BDT input variables are chosen so that the BDT output and the three-muon mass are uncorrelated in the mass range used in the analysis. A tight selection, following an initial cut on the BDT output, is applied to separate the signal from the background. After the optimal cut on the BDT output is found, a search is performed for an excess of events at the $\tau$ lepton mass above the expected background level.

The branching fraction is calculated as1where $N_\mathrm{s}$ is the number of observed events above the expected background level in a narrow region around the $\tau$ lepton mass,  is the detector acceptance times efficiency for the signal, and $N_{W\rightarrow \tau \nu }$ is the number of $\tau$ leptons produced via the $W\rightarrow \tau \nu$ channel (additional contributions to the $\tau$ lepton yield are estimated to be less than 3 %).

## The ATLAS detector

The ATLAS experiment [15] at the LHC is a multi-purpose particle detector with a forward-backward symmetric cylindrical geometry and a near $4\pi$ coverage in solid angle.^1^ It consists of an inner tracking detector surrounded by a thin superconducting solenoid providing a 2 T axial magnetic field, electromagnetic and hadronic calorimeters, and a muon spectrometer. The inner tracking detector (ID) covers the pseudorapidity range $|\eta | < 2.5$. It consists of silicon pixel, silicon microstrip, and transition radiation tracking detectors. Lead/liquid-argon (LAr) sampling calorimeters provide electromagnetic (EM) energy measurements with high granularity. A hadronic (iron/scintillator-tile) calorimeter covers the central pseudorapidity range ($|\eta | < 1.7$). The endcap and forward regions are instrumented with LAr calorimeters for EM and hadronic energy measurements up to $|\eta | = 4.9$.

The muon spectrometer (MS) comprises separate trigger and high-precision tracking chambers measuring the deflection of muons in a magnetic field generated by superconducting air-core toroids. The magnets’ bending power is in the range from 2.0 to 7.5 T m. The muon tracking chambers cover the region $|\eta | < 2.7$ with three layers of monitored drift tubes, complemented by cathode-strip chambers in the forward region, where the background is highest. The muon trigger system covers the range $|\eta | < 2.4$ with resistive-plate chambers in the barrel, and thin-gap chambers in the endcap regions.

A three-level trigger system is used to select events. The first-level trigger is implemented in hardware and uses a subset of the detector information to reduce the accepted rate to at most 75 kHz. This is followed by two software-based trigger levels that together reduce the accepted event rate to 400 Hz on average.

During the data-taking period, there were no dedicated triggers implemented for this analysis. A combination of seven muon triggers is used, where all triggers are constructed from at least two trigger objects. A detailed discussion of the trigger is given in Sect. 4.

## Simulation and data samples

The results presented here are based on proton–proton collision data at a centre-of-mass energy of $\sqrt{s} = 8$ TeV, collected by the ATLAS detector at the LHC during 2012. Data samples corresponding to an integrated luminosity of 20.3 fb$^{-1}$ are used. Selected data events are required to have all relevant components of the ATLAS detector in good working condition.

The Monte Carlo (MC) simulated $W\rightarrow \tau \nu \rightarrow (3\mu )\nu$ signal sample is produced by the Pythia8  [16] event generator (version 8.175) using the AU2 [17] set of tuned parameters and the MSTW2008LO parton distribution function (PDF) set [18]. This signal sample is modelled using $W\rightarrow \tau \nu$ production where the $\tau$ lepton is forced to decay isotropically into three muons as in previous searches for this mode [7–10]. The detector response is modelled using GEANT4 [19, 20]. The number of $\tau$ leptons produced in the 2012 dataset via the $W\rightarrow \tau \nu$ channel appearing in Eq. (1), is estimated by scaling the ATLAS measurement of the $W\rightarrow \ell \nu$ cross-section at $\sqrt{s}=7$ TeV [21] to 8 TeV using the ratio of the 8 TeV to 7 TeV NNLO cross-section calculations ($\sigma _\mathrm{theory}^\mathrm{8~TeV} = 12.18\pm 0.61$ nb and $\sigma _\mathrm{theory}^\mathrm{7~TeV} = 10.46\pm 0.52$ nb) and multiplying by the 8 TeV integrated luminosity. The result is $N_{W\rightarrow \tau \nu } =(2.41\pm 0.08) \times 10^8$, taking into account the uncertainty reported in Ref. [21] and the uncertainty in the 7 and 8 TeV luminosities. For the selection applied in the analysis, the contamination from other sources of $\tau$ leptons, such as $Z\rightarrow \tau \tau$ or HF processes, is less than 3 % and is therefore neglected. The background is estimated using data as discussed in Sect. 5.5.

## Trigger and reconstruction

To maximise the signal acceptance times efficiency, events are required to pass at least one of seven triggers. These are six multi-muon triggers and one dimuon plus $E_\mathrm{T}^\mathrm{miss}$ trigger. The software-based trigger thresholds used for the muons range from 4 to 18 GeV in transverse momentum while the $E_\mathrm{T}^\mathrm{miss}$ threshold is 30 GeV. The trigger efficiency for simulated signal events within the muon-trigger acceptance (three generator-level muons with $p_\mathrm{T} >2.5$ GeV and $|\eta |<2.4$) is ${\sim } 31~\%$ for the combination of all triggers used in the analysis. To evaluate the trigger performance in the region where the muons have a small angular separation, as is typical for the signal, a tag-and-probe study is performed using data events containing high-momentum $J/\psi \rightarrow \mu \mu$ candidates. For this study, the data are collected using a single-muon baseline trigger with a $p_\mathrm{T}$ threshold of 18 GeV. Single-muon efficiencies are measured separately for the different thresholds which define the six multi-muon triggers. Each multi-muon trigger efficiency is calculated as the product of the single-muon efficiencies. Correction factors are applied to account for the limited performance of the trigger system in identifying a pair of muons as two muon-trigger objects. At small angular separations ($\Delta R \lesssim 0.2$), where most of the signal is expected and where these limitations are most pronounced, these corrections must be taken into account. These factors are measured from the efficiency to identify two independent muon-trigger objects for different $\Delta R$ values between the tag- and the probe-muon. The total efficiency of the seven triggers is calculated considering correlations between any of the triggers. The trigger efficiency, measured from the data, is compared to the one measured in simulated $J/\psi$ events for the seven different triggers separately and jointly. Agreement between data and MC simulation was found to be within 11 % for all relevant values of $\Delta R$ and $p_\mathrm{T}$, where the largest difference comes from events where the $\Delta R$ separation is smallest. The systematic uncertainty on  due to the trigger is therefore taken to be 11 %.

The approach for measuring the muon reconstruction efficiency is similar to that used to measure the trigger efficiency. While the trigger efficiency is measured with respect to muon reconstruction as the baseline, the reconstruction efficiency is measured with respect to ID tracking, which in turn is close to $100~\%$ efficient [22]. Small deviations from the assumed value for ID tracking efficiency have a negligible impact on this measurement. The tag-and-probe procedure is performed using muons as tags and ID tracks as probes. The baseline sample for the reconstruction efficiency measurement includes a large number of non-muon tracks, which must be subtracted. This is done in bins of probe-track $p_\mathrm{T}$ ($p_\mathrm{T}^\mathrm{trk}$) and bins of the angular separation between the tag-muon and the probe-track, $\Delta R_{\mu + \mathrm{trk}}$. To describe the $J/\psi$ peak and the background, a small range in tag-muon plus probe-track invariant mass, $m_{\mu +\mathrm{trk}}\in [2600,3500]$ MeV, is fit to a double Gaussian function plus an exponential function and a second-order polynomial. In each $p_\mathrm{T}^\mathrm{trk}$ or $\Delta R_{\mu + \mathrm{trk}}$ bin, the ratio of the $J/\psi$ peak component integral to the full shape ($J/\psi$ plus background) integral is used as a weight to correct the $p_\mathrm{T}^\mathrm{trk}$ or $\Delta R_{\mu + \mathrm{trk}}$ shape itself. This is done separately for the probe-track distributions (denominators) and the muon-matched probe-track distributions (numerators). The ratio of the above two weighted distributions is defined as the reconstruction efficiency per $p_\mathrm{T}^\mathrm{trk}$ or $\Delta R_{\mu + \mathrm{trk}}$ bin. The efficiency measured with this approach in data is compared with the one from simulation and the difference at small $\Delta R_{\mu + \mathrm{trk}}$ results in an uncertainty of 13.1 % per event.

## Analysis procedure

The analysis procedure is divided into four steps. First, events containing three high-quality muon objects with a combined invariant mass of less than 2.5 GeV are selected. These muons are required to originate from a common vertex. Second, a *loose* selection is applied to this sample to obtain a background sample that can be used to train the BDT, which is constructed using the TMVA toolkit [23]. The *loose* selection cuts (using a number of vertex quantities as well as kinematic quantities) are chosen to obtain a large background sample for training, while rejecting background that is kinematically inconsistent with the signal. Before training the BDT, the data events are divided into three regions based on the three-muon mass value. These are the blinded region (which includes the signal region), a sideband region and a BDT training region as defined in Table 1. Third, a *tight* selection (tightening the *loose* selection with a few additional cuts) is applied while simultaneously placing an initial loose cut on the BDT score, denoted by $x{>}x_0$. The $x{>}x_0$ cut removes background-like events having a very low BDT score, while the *tight* selection further reduces the background in the blinded and sideband regions. Fourth, the background rejection as a function of the BDT cut is studied using data events in the sideband region passing the *tight* ${+}x{>}x_0$ selection. This allows to optimise the final cut on the BDT score, denoted by $x{>}x_1$. The statistical analysis is performed for the *tight* ${+}x{>}x_1$ selection.

**Table 1:** The different three-muon mass ranges used in the analysis

Region	Range in $m_{3\mu }$ [MeV]
Signal region	$[1713,1841]$
Blinded region	$[1690,1870]$
Sideband region	$[1450,1690]$ and $[1870,2110]$
Training region	$[750,1450]$ and $[2110,2500]$

The signal region (SR) is defined as an interval around the $\tau$ lepton mass with a half-width corresponding to twice the resolution of the three-muon mass, $\sigma _s = 32$ MeV, as obtained from the signal MC sample. The analysis was blinded in a slightly wider region to allow variation of the signal region definition. The signal MC sample is divided into two independent samples. One signal sample is used for the BDT training while the second signal sample is used for estimating the . The background in the signal region is estimated from a fit to the three-muon mass distribution in the sidebands (SB) using the *tight* ${+}x{>}x_0$ selection. This estimate is then scaled down to the final BDT score cut, $x_1$, using a fit to the BDT shape as explained below.

### Object selection

Muons are selected to have a transverse momentum greater than 2.5 GeV and are required to pass stringent requirements on the track quality and the associated hits in both the ID and the MS. Only combined ID+MS measurements of track parameters are used. Several matching criteria [22] are imposed to reject non-muon tracks (e.g. tracks from hadron decays in flight). The performance of muon identification is validated in two dedicated dimuon control regions. One region is populated with muons from $J/\psi \rightarrow \mu \mu$ decay (in $2850 < m_{2\mu } < 3350$ MeV), while the second region has an enhanced fraction of non-muon tracks (in events with $m_{2\mu } < 750$ MeV).

Events with at least three selected muons are considered. All possible three-muon combinations are used as inputs to a vertex fit. The primary vertex (PV) is also refitted after removing the three tracks. Due to the $\tau$ lepton lifetime, the three-muon vertex is often separated from the PV. The characteristics of the separation between the three-muon vertex and the PV are therefore used to distinguish signal from background. Particularly, the two projections of the three-muon vertex displacement with respect to the PV in the transverse plane are used; $L_{xy} {=}L_\mathrm{T}\cos \theta _{xy}$ and $a^{0}_{xy} {=}L_\mathrm{T}\sin \theta _{xy}$ where $L_\mathrm{T}$ is the transverse component of the vector connecting the PV and the three-muon vertex and $\cos \theta _{xy}{=}\frac{\vec {L}_\mathrm{T}\cdot \vec {p}_\mathrm{T}^{3\mu }}{L_\mathrm{T} p_\mathrm{T}^{3\mu }}$. The three-muon vertex fit probability, $p$-value, is also used (as calculated from the vertex fit $\chi ^2$ and degrees of freedom). After fitting all possible vertices, exactly one three-muon candidate is allowed per event, satisfying $m_{3\mu } <2500$ MeV and $|Q_{3\mu } |=1$ where $Q_{3\mu }$ is the sum of the charges of the three-muon tracks.

Jets are used to separate the signal from the multi-jet backgrounds (predominantly HF), where more hadronic activity is expected. The jets are reconstructed from topological clusters formed in the calorimeter using the anti-$k_t$ algorithm [24] with a radius parameter $R=0.4$. The jets are calibrated to the hadronic energy scale using energy- and $\eta$-dependent correction factors derived from simulation and with residual corrections from in situ measurements. A detailed description of the jet energy scale measurement and its systematic uncertainties can be found in Ref. [25]. Jets found within a cone of $\Delta R = 0.2$ around a selected three-muon candidate are removed. Jets are required to have $p_\mathrm{T} >30$ GeV and $|\eta |<2.8$; only the leading jet satisfying these criteria is considered. There is no veto of events with more than one jet satisfying these criteria. The leading jet and the three-muon momenta are summed vectorially to define $\vec {\Sigma } = \vec {p}_\mathrm{jet} + \vec {p}_{3\mu }$ with $\Sigma _\mathrm{T}$ being the magnitude of its transverse component. For events where there are no jets satisfying these criteria (the majority of events for the signal), $\vec {\Sigma }$ is simply $\vec {p}_{3\mu }$.

The $E_\mathrm{T}^\mathrm{miss}$ is calculated as the negative vector sum of the transverse momenta of all high-$p_\mathrm{T}$ objects reconstructed in the event, as well as a term for other activity in the calorimeter [26]. Clusters associated with electrons, hadronic $\tau$ lepton decays and jets are calibrated separately, with other clusters calibrated at the EM energy scale. This $E_\mathrm{T}^\mathrm{miss}$ is denoted hereafter by $E_\mathrm{T,cal}^\mathrm{miss}$. In addition, a track-based missing transverse momentum ($E_\mathrm{T,trk}^\mathrm{miss}$) is calculated as the negative vector sum of the transverse momenta of tracks with $|\eta |<2.5$, $p_\mathrm{T} >500$ MeV and associated with the primary vertex. Both the calorimeter-based and track-based measurements of the $E_\mathrm{T}^\mathrm{miss}$ are used.

Several kinematic variables are defined from the reconstructed objects listed above. Two transverse masses are defined using the three-muon transverse momentum ($p_\mathrm{T}^{3\mu }$) as $m_\mathrm{T} = \sqrt{2p_\mathrm{T}^{3\mu } E_\mathrm{T}^\mathrm{miss} (1-\cos \Delta \phi _{3\mu })}$, where $\Delta \phi _{3\mu }$ is the angle between the $E_\mathrm{T}^\mathrm{miss}$ and $p_\mathrm{T}^{3\mu }$ directions in the transverse plane. In these definitions, $E_\mathrm{T}^\mathrm{miss}$ can be either $E_\mathrm{T,cal}^\mathrm{miss}$ or $E_\mathrm{T,trk}^\mathrm{miss}$ to obtain $m_\mathrm{T}^\mathrm{cal}$ or $m_\mathrm{T}^\mathrm{trk}$ respectively. The $\Delta \phi _{3\mu }$ terms are $\Delta \phi _{3\mu }^\mathrm{cal}$ or $\Delta \phi _{3\mu }^\mathrm{trk}$ respectively. Similarly, the $\Delta \phi _{\Sigma _\mathrm{T}}$ variable is the angle between the $E_\mathrm{T}^\mathrm{miss}$ and $\Sigma _\mathrm{T}$ directions in the transverse plane. This adds two additional angles, $\Delta \phi _{\Sigma _\mathrm{T}}^\mathrm{cal}$ and $\Delta \phi _{\Sigma _\mathrm{T}}^\mathrm{trk}$ for $E_\mathrm{T,cal}^\mathrm{miss}$ and $E_\mathrm{T,trk}^\mathrm{miss}$ respectively. These $\Delta \phi _{\Sigma _\mathrm{T}}$ variables provide good separation when a hard jet is found and thus $\Sigma _\mathrm{T}$ deviates from $p_\mathrm{T}^{3\mu }$ in magnitude and direction.

### Loose event selection

After the three-muon candidates are formed from the selected muons, a *loose* event selection is performed, maintaining a signal efficiency of about 80 % while rejecting about 95 % of the background. This *loose* selection includes cuts on the displacement of the vertex from the PV, requirements on the three-muon kinematics and on the presence of other tracks (track isolation), and requirements on quantities involving $E_\mathrm{T,cal}^\mathrm{miss}$ and $E_\mathrm{T,trk}^\mathrm{miss}$. The *loose* selection comprises the following requirements:

- The $L_{xy}$ significance, $S(L_{xy}) {=}L_{xy}/\sigma _{L_{xy}}$, must satisfy $-10{<}S(L_{xy}) {<}50$, where $\sigma _{L_{xy}}$ is the uncertainty in the $L_{xy}$.
- The $a^{0}_{xy}$ significance, $S(a^{0}_{xy}) {=}a^{0}_{xy}/\sigma _{a^{0}_{xy}}$, must satisfy $S(a^{0}_{xy}) {<}25$, where $\sigma _{a^{0}_{xy}}$ is the uncertainty in $a^{0}_{xy}$.
- The three-muon track-fit probability product, $\mathcal {P}_\mathrm{trks} = p_1\times p_2\times p_3$ (where $p_i$ is the track fit $p$-value of track *i*), must satisfy $\mathcal {P}_\mathrm{trks} >10^{-9}$.
- The three-muon transverse momentum must satisfy $p_\mathrm{T}^{3\mu } >10$ GeV.
- The missing transverse energies, $E_\mathrm{T,cal}^\mathrm{miss}$ and $E_\mathrm{T,trk}^\mathrm{miss}$, must both satisfy $10<E_\mathrm{T}^\mathrm{miss} < 250$ GeV.
- The transverse masses, $m_\mathrm{T}^\mathrm{cal}$ and $m_\mathrm{T}^\mathrm{trk}$, must both satisfy $m_\mathrm{T}>20$ GeV.
- The three-muon track isolation is obtained from the sum of the $p_\mathrm{T}$ of all tracks with $p_\mathrm{T}^\mathrm{trk}>500$ MeV in a cone of $\Delta R^{3\mu }_\mathrm{max} +0.20$ (and $\Delta R^{3\mu }_\mathrm{max} +0.30$) around the three-muon momentum while excluding its constituent tracks; it must satisfy $\Sigma p_\mathrm{T}^\mathrm{trk}(\Delta R^{3\mu }_\mathrm{max} +0.20)/p_\mathrm{T}^{3\mu } <0.3$ (and $\Sigma p_\mathrm{T}^\mathrm{trk}(\Delta R^{3\mu }_\mathrm{max} +0.30)/p_\mathrm{T}^{3\mu } <1$). The largest separation, $\Delta R^{3\mu }_\mathrm{max}$, between any pair of the three-muon tracks is on average 0.07 for the signal.

The loose cuts on the significances, $S(L_{xy})$ and $S(a^{0}_{xy})$, are applied to allow the three-muon vertex to be separated from the PV, while still being compatible with the $\tau$ lepton lifetime. The requirement on $\mathcal {P}_\mathrm{trks}$ imposes a goodness-of-fit criterion on the three-muon candidate. This value is based on an examination of signal-like events found in the sideband region in the data. As this is not the only quality requirement imposed on the individual muon objects, it is kept loose in this part of the selection. The efficiency for this cut to select signal events is ${\sim } 98~\%$, while it is rejecting ${\sim } 13~\%$ of the background events. The kinematic and the isolation variables are very effective in separating the *W* boson properties of the signal from the HF and the light-flavour multi-jet background, which tend to be non-isolated and with low values of $p_\mathrm{T}$, $E_\mathrm{T}^\mathrm{miss}$ and $m_\mathrm{T}$. The associated cuts remain very loose in this part of the selection to ensure that the sample sizes are large enough for the BDT training.

### Multivariate analysis

The events passing the *loose* selection described above are used as input to the BDT training. There are 6649 events passing the loose selection in the signal MC sample (out of $10^5$), where 6000 of these events are used for the BDT training and the rest are used for testing the BDT output. Similarly, the number of data events passing the *loose* selection in the training region is 4672, where 4000 of these events are used for the BDT training. The BDT input variables include kinematic distributions, track and vertex quality discriminants, vertex geometry parameters, and isolation. The following variables (sorted by their importance ranking) are used as inputs to the BDT:

1. The calorimeter-based transverse mass, $m_\mathrm{T}^\mathrm{cal}$.
2. The track-based missing transverse momentum, $E_\mathrm{T,trk}^\mathrm{miss}$.
3. The isolation variable, $\Sigma p_\mathrm{T}^\mathrm{trk}(\Delta R^{3\mu }_\mathrm{max} +0.20)/p_\mathrm{T}^{3\mu }$.
4. The transverse component of the vector sum of the three-muon and leading jet momenta, $\Sigma _\mathrm{T}$.
5. The track-based transverse mass, $m_\mathrm{T}^\mathrm{trk}$.
6. The difference between the $E_\mathrm{T,cal}^\mathrm{miss}$ and $E_\mathrm{T,trk}^\mathrm{miss}$ directions, $\Delta \phi _\mathrm{trk}^\mathrm{cal}$.
7. The calorimeter-based missing transverse momentum, $E_\mathrm{T,cal}^\mathrm{miss}$.
8. The track-based missing transverse momentum balance $p_\mathrm{T}^{3\mu }/E_\mathrm{T,trk}^\mathrm{miss}-1$.
9. The difference between the three-muon and $E_\mathrm{T,cal}^\mathrm{miss}$ directions, $\Delta \phi _{3\mu }^\mathrm{cal}$.
10. Three-muon vertex fit probability, $p$-value.
11. The three-muon vertex fit $a^{0}_{xy}$ significance, $S(a^{0}_{xy})$.
12. The track fit probability product, $\mathcal {P}_\mathrm{trks}$.
13. The three-muon transverse momentum, $p_\mathrm{T}^{3\mu }$.
14. The number of tracks associated with the PV (after refitting the PV while excluding the three-muon tracks), $N_\mathrm{trk}^\mathrm{PV}$.
15. The three-muon vertex fit $L_{xy}$ significance, $S(L_{xy})$.
16. The calorimeter-based missing transverse momentum balance, $p_\mathrm{T}^{3\mu }/E_\mathrm{T,cal}^\mathrm{miss}-1$.

This configuration was found to give the optimal balance between background rejection and signal efficiency.

The $\Sigma _\mathrm{T}$ variable is introduced to avoid vetoing events with at least one jet fulfilling the requirements listed in Sect. 5.1. Although the majority of signal events do not have jets, it is found that keeping such events increases the  by ${\sim } 15~\%$ and also ultimately leads to better rejection power, owing to the significantly larger training and sideband samples. The variables $\Delta \phi _\mathrm{trk}^\mathrm{cal}$, $\Delta \phi _{3\mu }^\mathrm{cal}$, $p_\mathrm{T}^{3\mu }/E_\mathrm{T,trk}^\mathrm{miss}-1$, $p_\mathrm{T}^{3\mu }/E_\mathrm{T,cal}^\mathrm{miss}-1$ and $N_\mathrm{trk}^\mathrm{PV}$ are complementary to the $E_\mathrm{T}^\mathrm{miss}$-related variables used in the *loose* selection as well as here. These variables are also very effective in distinguishing the $W\rightarrow \tau \nu$ production of the signal from the HF and light-flavour multi-jet background. The vertex $p$-value is a variable complementary to the $S(L_{xy})$ and $S(a^{0}_{xy})$ variables used in the *loose* selection as well as here. The HF and light-flavour multi-jet backgrounds have mostly random combinations of selected muon objects which do not originate from the same vertex. This variable peaks at very low values for the background while for the signal it is distributed uniformly and thus provides excellent separation.

After training the BDT with data events from the training region and signal MC events from the first signal MC sample, the BDT response is calculated for the data events in the sidebands and for events in the second signal sample. The BDT score, *x*, ranges between $-$1 and $+$1. Events with a very low BDT score, within $-1\le x\le -0.9$ are removed from further consideration, defining $x_0\equiv -0.9$.

In order to assess potential modelling problems in the signal MC sample, the BDT input distributions and the BDT response are validated against single-muon data. These data contain mainly $W\rightarrow \mu \nu$ events with a small fraction (${<}10~\%$) of background. The single-muon selection is formulated to be as close as possible to the main analysis selection where the differences are mostly driven by the different triggers used (one single-muon trigger with no isolation requirement and with a threshold of 24 GeV is used in the validation) and the exclusion of variables which do not have equivalents in the $W\rightarrow \mu \nu$ case, e.g. the three-muon vertex variables. The training samples used for this validation study, for both data and signal, are the same samples as used in the main analysis, constructed with the same *loose* selection as described in the previous section. All input variables are used for the training, excluding the $p$-value, $S(L_{xy})$, $S(a^{0}_{xy})$ and $\mathcal {P}_\mathrm{trks}$, which cannot be calculated in a single-muon ($W\rightarrow \mu \nu$) selection. The resulting BDT setup is hereafter referred to as “partial BDT”. After training the partial BDT, the response is tested on the second signal sample and on the single-muon data, using the single-muon selection and where the three muon objects in the signal sample are treated as one object (muon). The $N_\mathrm{trk}^\mathrm{PV}$ distribution of the signal sample is also modified by subtracting two tracks to reflect the difference with respect to a single-muon selection. The responses in data and simulation are compared and are found to agree within 10 % throughout most of the phase-space for all variables. The ratio of the partial BDT responses for the single-muon data and signal MC events is used as an event weight while applying the full selection and calculating the weighted  as described in the next sections. The difference between the weighted and unweighted  is found to be 4 % and is taken as a modelling uncertainty.

Any variable which may bias the BDT response by only selecting events very close to the $\tau$ lepton mass is not included in the BDT input list. The distribution of the three-muon mass has been examined in several bins of *x* above $x_0$ using both the *loose* and the *tight* samples, where no hint of potential peaking background around the $\tau$ lepton mass has been found. In addition, the shape of the three-muon mass distribution has been found to be insensitive to the BDT cut, as expected given the small correlation coefficient between *x* and $m_{3\mu }$, which is found to be about $-$0.05.

### Tight event selection

Additional *tight* cuts are applied after the BDT training and the application of the $x{>}x_0$ cut on the BDT score. The following requirements are tightened or added:

- A number of the *loose* requirements are tightened, namely $\mathcal {P}_\mathrm{trks} >8\times 10^{-9}$, $m_\mathrm{T}^\mathrm{cal} >45$ GeV, $m_\mathrm{T}^\mathrm{trk} >45$ GeV and $1{<}S(L_{xy}) {<}50$.
- Three-muon vertex fit probability must have $p\mathrm{-value} >0.2$.
- The angle between the $\Sigma _\mathrm{T}$ and $E_\mathrm{T,cal}^\mathrm{miss}$ ($E_\mathrm{T,trk}^\mathrm{miss}$) directions is required to be $\Delta \phi _{\Sigma _\mathrm{T}}^\mathrm{cal} >2$ ($\Delta \phi _{\Sigma _\mathrm{T}}^\mathrm{trk} >2$).
- The same-charge two-muon mass, $m_\mathrm{SS}$, and opposite-charge two-muon mass, $m_\mathrm{OS1}$ or $m_\mathrm{OS2}$, satisfy $m_\mathrm{SS} >300$ MeV, $m_\mathrm{OS1} >300$ MeV and $m_\mathrm{OS2} >300$ MeV, where $m_\mathrm{OS1}$ ($m_\mathrm{OS2}$) is the mass of the two opposite-charge muon pairs with the highest (second highest) summed scalar $p_\mathrm{T}$ among the three muons.
- The event is rejected if $|m_\mathrm{OS}-m_\omega |<50$ MeV or $|m_\mathrm{OS}-m_\phi |<50$ MeV if either of the $p_\mathrm{T}^{3\mu }$, the $E_\mathrm{T,cal}^\mathrm{miss}$ or the $E_\mathrm{T,trk}^\mathrm{miss}$ is lower than 35 GeV.
- The event is rejected if $|m_\mathrm{OS}-m_\phi |<50$ MeV if $|m_{3\mu }-m_{D_s} |<100$ MeV.

In the above notation, $m_\mathrm{OS}$ is $m_\mathrm{OS1}$ or $m_\mathrm{OS2}$ and $m_\omega$, $m_\phi$ and $m_{D_s}$ are the masses of the $\omega$, $\phi$ and $D_s$ mesons respectively, taken from Ref. [2].

The requirement on the three-muon vertex fit probability is applied in order to ensure a high-quality fit. The cuts on $\Delta \phi _{\Sigma _\mathrm{T}}^\mathrm{cal}$ ($\Delta \phi _{\Sigma _\mathrm{T}}^\mathrm{trk}$) are applied in order to further suppress the HF and multi-jet background where the three-muon candidate is typically produced within or near a jet.

The first two-muon mass requirement is applied to suppress candidates originating from one prompt muon object and two muons from a converted photon. The second requirement on the two-muon masses is applied to prevent the low-mass mesons, $\rho /\omega$ and $\phi$, from entering into the region close to the $\tau$ lepton mass when combined with an additional track. In the selected three-muon event sample, these resonances appear as two clear peaks in the mass distribution of oppositely charged muon pairs in data. Since the resonances lie in the middle of the signal distribution, the low-$p_\mathrm{T}$ and $E_\mathrm{T}^\mathrm{miss}$ requirement ensures that these can still be distinguished from the signal, and thus it removes the resonances while still maintaining a high enough signal efficiency. Finally, the last requirement is applied to remove a potential $D_s \rightarrow \pi +\phi (\mu \mu )$ contamination from the high-mass sideband. The cuts listed above comprise the *tight* selection where the *tight* ${+}x{>}x_0$ selection is used to estimate the background for any cut on *x* above $x_0$.

**Fig. 1:**
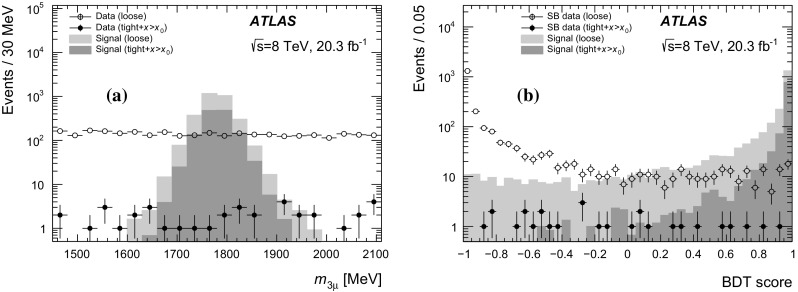
The three-muon mass distribution in **a** and the BDT score in **b**. The BDT score distribution of the data is shown for the sideband region. The *loose* data are shown as hollow circles, while the *loose* signal MC events are shown as *light solid grey area*. The *tight* ${+}x{>}x_0$ data are shown as the *solid black circles*, while the *tight* ${+}x{>}x_0$ signal MC events are shown as the *dark solid grey area*. The area of the signal MC shapes is normalised to the area of the *loose* data shapes and the relative normalisation difference between the *loose* and the *tight* ${+}x{>}x_0$ MC signal distributions prior to the normalisation is maintained. For illustration, the signal is not constrained to the SR

**Fig. 2:**
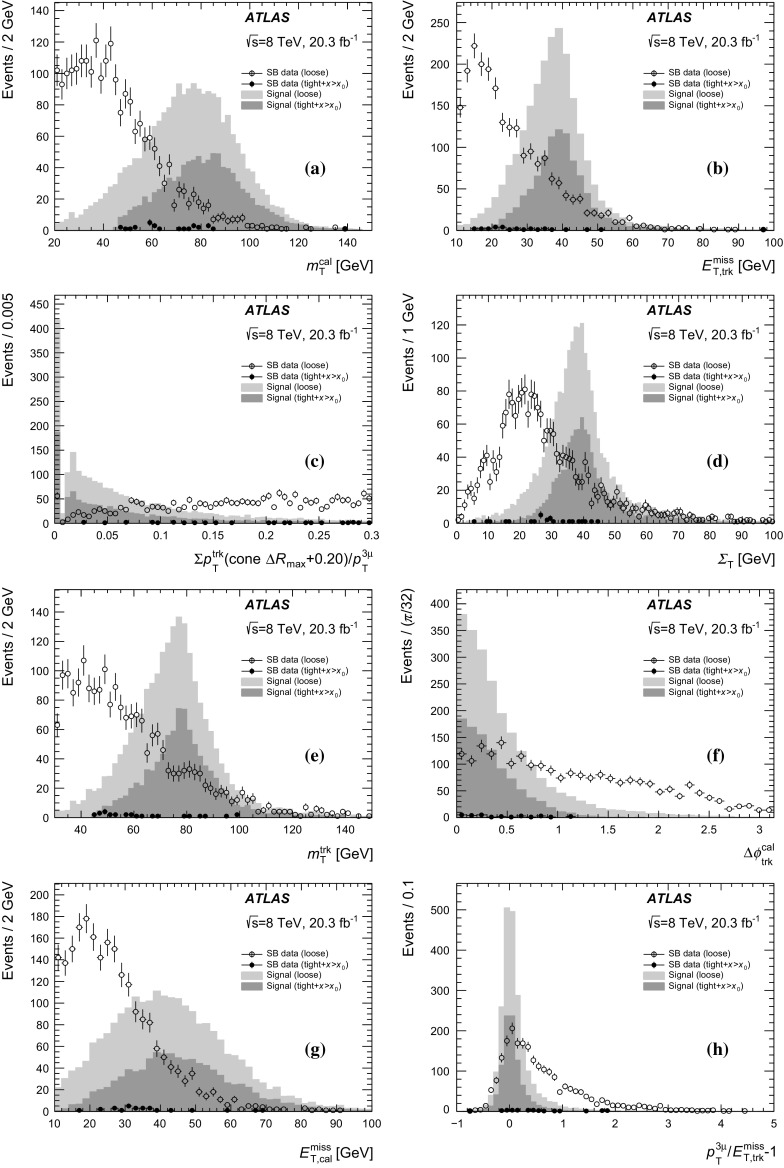
The BDT inputs ranked 1–8. $m_\mathrm{T}^\mathrm{cal}$ in **a**, $E_\mathrm{T,trk}^\mathrm{miss}$ in **b**, $\Sigma p_\mathrm{T}^\mathrm{trk}(\Delta R^{3\mu }_\mathrm{max} +0.20)/p_\mathrm{T}^{3\mu }$ in **c**, $\Sigma _\mathrm{T}$ in **d**, $m_\mathrm{T}^\mathrm{trk}$ in **e**, $\Delta \phi _\mathrm{trk}^\mathrm{cal}$ in **f**, $E_\mathrm{T,cal}^\mathrm{miss}$ in **g** and $p_\mathrm{T}^{3\mu }/E_\mathrm{T,trk}^\mathrm{miss}-1$ in **h**. The *loose* data in the sidebands are shown as *hollow circles*, while the *loose* signal MC events are shown as *light solid grey area*. The *tight* ${+}x{>}x_0$ data in the sidebands are shown as the *solid black circles*, while the *tight* ${+}x{>}x_0$ signal MC events are shown as the *dark solid grey area*. The area of the signal MC shapes is normalised to the area of the *loose* data shapes and the relative normalisation difference between the *loose* and the *tight* ${+}x{>}x_0$ MC signal distributions prior to the normalisation is maintained. For illustration, the signal is not constrained to the SR

**Fig. 3:**
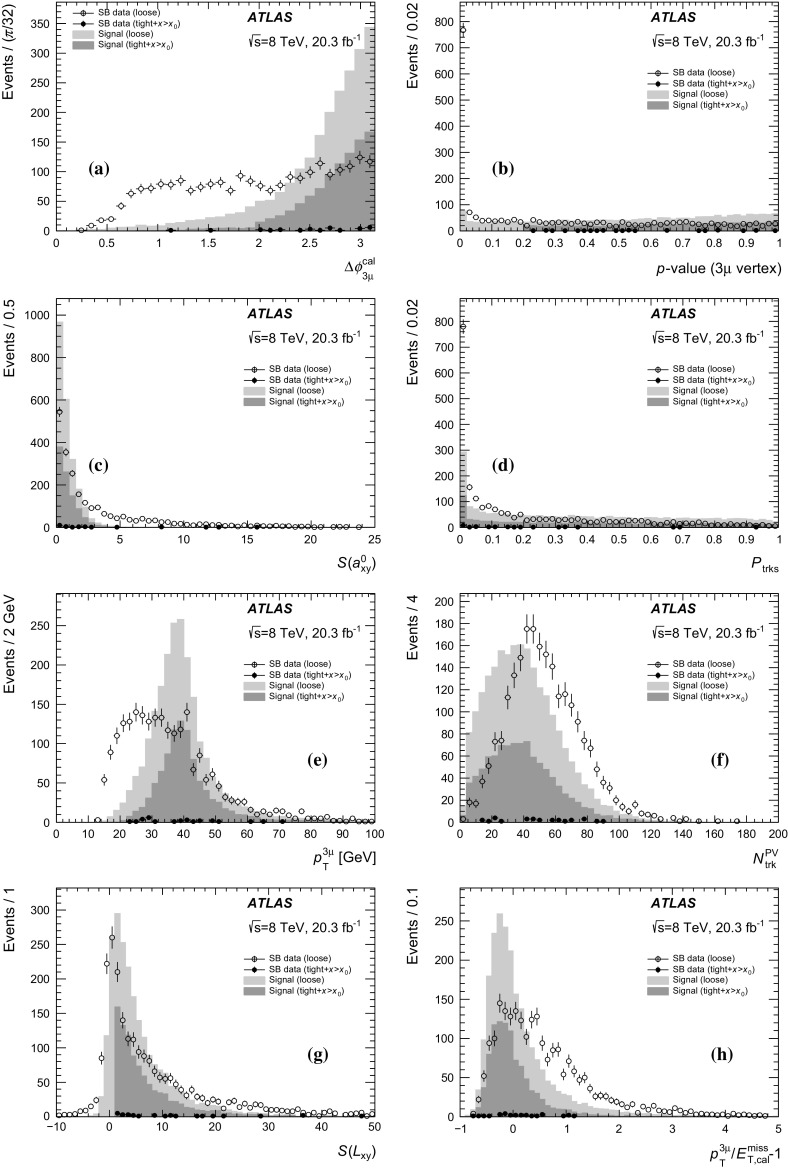
The BDT inputs ranked 9–16. $\Delta \phi _{3\mu }^\mathrm{cal}$ in **a**, $p$-value in **b**, $S(a^{0}_{xy})$ in **c**, $\mathcal {P}_\mathrm{trks}$ in **d**, $p_\mathrm{T}^{3\mu }$ in **e**, $N_\mathrm{trk}^\mathrm{PV}$ in **f**, $S(L_{xy})$ in **g** and $p_\mathrm{T}^{3\mu }/E_\mathrm{T,cal}^\mathrm{miss}-1$ in **h**. The *loose* data in the sidebands are shown as *hollow circles*, while the *loose* signal MC events are shown as *light solid grey area*. The *tight* ${+}x{>}x_0$ data in the sidebands are shown as the *solid black circles*, while the *tight* ${+}x{>}x_0$ signal MC events are shown as the *dark solid grey area*. The area of the signal MC shapes is normalised to the area of the *loose* data shapes and the relative normalisation difference between the *loose* and the *tight* ${+}x{>}x_0$ MC signal distributions prior to the normalisation is maintained. For illustration, the signal is not constrained to the SR

**Fig. 4:**
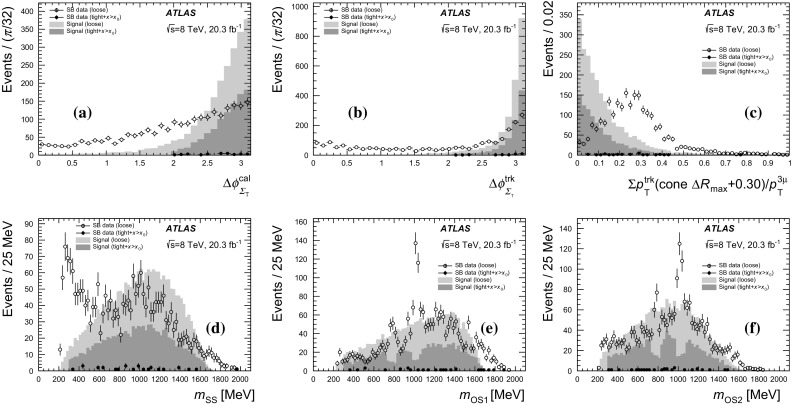
The complementary variables used in the *loose* or *tight* selection but not as inputs for the BDT. $\Delta \phi _{\Sigma _\mathrm{T}}^\mathrm{cal}$ in **a**, $\Delta \phi _{\Sigma _\mathrm{T}}^\mathrm{trk}$ in **b**, $\Sigma p_\mathrm{T}^\mathrm{trk}(\Delta R^{3\mu }_\mathrm{max} +0.30)/p_\mathrm{T}^{3\mu }$ in **c**, $m_\mathrm{SS}$ in **d**, $m_\mathrm{OS1}$ in **e** and $m_\mathrm{OS2}$ in **f**. The *loose* data in the sidebands are shown as *hollow circles*, while the *loose* signal MC events are shown as *light solid grey area*. The *tight* ${+}x{>}x_0$ data in the sidebands are shown as the *solid black circles*, while the *tight* ${+}x{>}x_0$ signal MC events are shown as the *dark solid grey area*. The area of the signal MC shapes is normalised to the area of the *loose* data shapes and the relative normalisation difference between the *loose* and the *tight* ${+}x{>}x_0$ MC signal distributions prior to the normalisation is maintained. For illustration, the signal is not constrained to the SR

Figure 1 shows the three-muon mass distribution and the BDT response distribution. Figures 2 and 3 show the distributions of the BDT inputs sorted by the separation rank as reported by TMVA during the BDT training. Figure 4 shows the distributions of the complementary variables which are used in the *loose* or *tight* selection but not in the BDT.

### Background estimation

The events passing the *tight* ${+}x{>}x_0$ selection are used to estimate the expected number of background events in the signal region for higher cuts on *x* as described below.

**Fig. 5:**
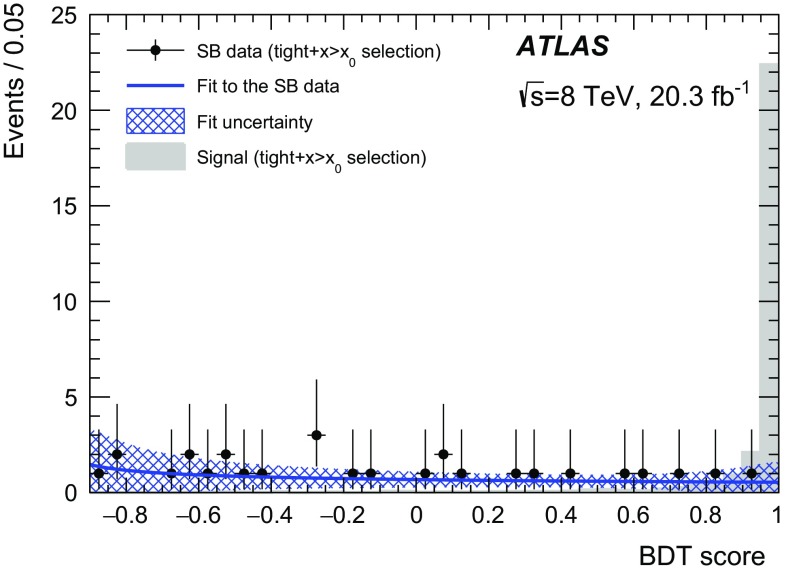
The distribution of the BDT score of the data in the sideband region (SB) for the *tight* ${+}x{>}x_0$ selection. The *line* shows the result of a fit to the BDT score distribution, while the *hatched area* shows the uncertainty in the fit due to the SB range definition, the $x_0$ cut location and the fit function choice. The *solid grey area* shows the signal shape (obtained from MC simulation), normalised to the area of the data for the *tight* ${+}x{>}x_0$ selection. For illustration, the signal is not constrained to the SR

The signal MC and sideband data BDT responses are shown in Fig. 5 after the *tight* ${+}x{>}x_0$ selection. The distinct shapes illustrate the power of the method in separating the background from the signal. The analytical function also shown in Fig. 5 is a result of a fit to the sideband data, excluding the blinded region, using an unbinned maximum-likelihood estimator. The fit function used is $a_0 + a_1(x+1)^{a_2}+a_3(x+1)^{a_4}$, where $a_i$ are the free fit parameters. The parameter $a_2$ is required to be negative while the other are required to be non-negative. This function can exhibit rising behaviour at both ends of the *x* distribution ($x\rightarrow \pm 1$) and it is used to scale the quantities measured in $x{>}x_0$ to the corresponding quantities in $x{>}x_1$ as explained below.

**Fig. 6:**
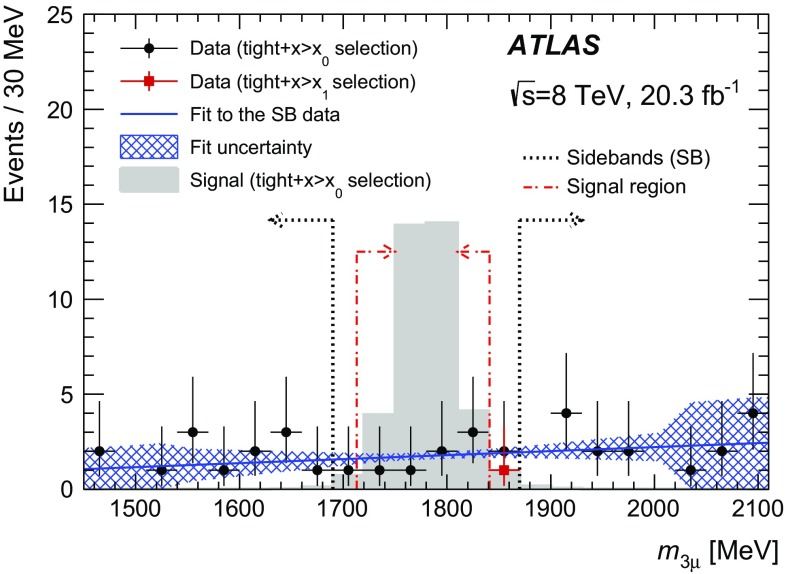
The three-muon mass distribution in the range [1450, 2110] MeV shown for the *tight* ${+}x{>}x_0$ selection by *solid black circles* and for the *tight* ${+}x{>}x_1$ selection by the *solid red square*. The sideband and signal regions are indicated by the *arrows*. The *tight* ${+}x{>}x_0$ data are fit in the two sidebands simultaneously, excluding the events in the blinded region. The *hatched area* shows the uncertainty in the fit due to the SB range definition, the $x_0$ cut location and the fit function choice. The *solid grey area* shows the signal shape (obtained from MC simulation), normalised to the area of the data for the *tight* ${+}x{>}x_0$ selection

The three-muon mass distribution of the *tight* ${+}x{>}x_0$ data is fit simultaneously in the two sidebands to a second-order polynomial in $m_{3\mu }$ while excluding the blinded region. This is also done with an unbinned maximum-likelihood estimator. The integral of the resulting fit function in the signal region gives the expected number of background events, $N_\mathrm{b}(x_0)$ in the signal region before applying the final $x_1$ cut. The statistical uncertainty of $N_\mathrm{b}(x_0)$ is calculated by scaling the statistical error in the number of events in the sidebands, according to the ratio of analytical integrals in the signal region and sidebands. Figure 6 shows the three-muon mass distribution in the sidebands for the *tight* ${+}x{>}x_0$ selection as black points together with the fit result. The signal is also shown for reference, scaled up arbitrarily to match the scale of the data.

For any $x_1$ cut value above $x\sim 0.6$, where most of the signal is expected, the estimated $N_\mathrm{b}(x_0)$ in the signal region can be then scaled down according to the ratio of the integrals of the BDT analytical function above and below this cut. This ratio is denoted hereafter by . The extrapolation procedure can be written as  where $N_\mathrm{b}(x_1)$ is estimated in the signal region for $x{>}x_1$.

### Uncertainties and optimisation

The sources of systematic uncertainty associated with the extrapolation procedure in the background estimation are the BDT and sideband fit function choice, the definition of the sideband ranges and the definition of $x_0$. To estimate this uncertainty, each of these definitions and choices is varied individually while calculating $N_\mathrm{b}(x_0)$ and . For each fit function (BDT and sideband), different parameterisations are considered. In addition, to construct the variation of the *tight* ${+}x{>}x_0$ sample with which the two fits are performed, nine different sideband range variations and ten different $x_0$ variations are used. The fits, and consequently also the extrapolation procedure, are found to be stable against these variations. The dominant uncertainty component is the impact on  of varying the sideband ranges definition. The differences from the nominal values of  and $N_\mathrm{b}(x_0)$ are summed in quadrature and are translated to uncertainties in $N_\mathrm{b}(x_1)$. The systematic uncertainty associated with the extrapolation procedure used to obtain $N_\mathrm{b}(x_1)$ increases with $x_1$ from ${\sim } 45~\%$ at $x_1=0.6$ to ${\sim } 80~\%$ at $x_1\simeq 1$. The statistical uncertainty of $N_\mathrm{b}(x_1)$ is ${\sim } 19~\%$, independent of $x_1$.

The systematic uncertainty in the signal acceptance times efficiency has contributions from reconstruction (13.1 %), trigger (11 %) and MC modelling (4 %) as discussed in the previous sections. In addition, there is a small (2.1 %) contribution due to jet and $E_\mathrm{T}^\mathrm{miss}$ calibration. The number of $\tau$ leptons produced via the $W\rightarrow \tau \nu$ channel and its uncertainty (3.9 %) are estimated as described in Sect. 3. These uncertainties are independent of $x_1$ in the range of interest.

The BDT cut is optimised by minimising the expected upper limit on the branching fraction given in Eq. (1), where $N_\mathrm{s}$ becomes the upper limit on the number of observed events above the expected background level in a narrow region around the $\tau$ lepton mass. The procedure is performed by varying $x_1$ between 0.6 and 1.0 in steps of 0.001 while extracting $N_\mathrm{b}(x_1)$ and its associated errors as explained above. To obtain the upper limit on $N_\mathrm{s}$ for each $x_1$ cut, a single-bin counting experiment is performed using the HistFitter [27] statistical framework, supplied with $N_\mathrm{b}(x_1)$ and its uncertainties. For compatibility with previous searches, the limit on $N_\mathrm{s}$ and on $\mathrm{Br}(\tau \rightarrow 3\mu )$ is given at 90 % confidence level (CL). In each iteration,  is calculated for the specific $x_1$ cut using a signal sample that is different from the one used for the BDT training.

During the iterative optimisation process, the extrapolation of the number of events in the sideband region to high $x_1$ cuts using the BDT shape is tested against a cut-and-count procedure. The two procedures are found to agree very well within the uncertainties, and the extrapolation procedure gives a more conservative result throughout the examined $x_1$ range. The resulting optimal cut is at $x_1=0.933$.

## Results

Figure 6 shows the three-muon mass distributions in the full mass range, including the blinded region, for the *tight* ${+}x{>}x_1$ selection in red squares. Only one event with a three-muon mass of 1860 MeV survives the selection in the full mass range (sideband and blinded regions). This event is found in the range between the signal region and the right sideband region and it does not affect the background estimation or the observation in the signal region.

The event counts entering the different regions at the different steps of the analysis for signal and data are given in Table 2.

**Table 2:** [Event counts]The event count for the different steps of the analysis in the sideband and signal regions. The signal sample used to evaluate the  has $2\times 10^5$ events

Phase	Data SB	Data SR	Signal MC SR [out of $2\times 10^5$]
*loose*	2248	580	12672
*loose* ${+}x{>}x_0$	736	203	12557
*tight*	42	9	5503
*tight* ${+}x{>}x_0$	28	7	5501
*tight* ${+}x{>}x_1$	0	0	4616

The signal acceptance times efficiency is calculated from the second signal MC sample after applying the full *tight* ${+}x{>}x_1$ selection. This selection corresponds to . With this selection, the expected background yield is $N_\mathrm{b}(x_1) =0.193 \pm 0.131_\mathrm{syst} \pm 0.037_\mathrm{stat}$. The systematic uncertainty on  is dominated by the uncertainties in the reconstruction and trigger efficiency measurements. The systematic uncertainty on $N_\mathrm{b}(x_1)$ is dominated by the uncertainty in the extrapolation of the background from the *tight* ${+}x{>}x_0$ selection to the *tight* ${+}x{>}x_1$ selection.

The systematic uncertainties in $N_\mathrm{b}$ are taken into account when calculating the limit on the number of signal events, $N_\mathrm{s}$, via one nuisance parameter. The systematic uncertainties in the product  are summed in quadrature and taken into account as the uncertainty in the signal via one nuisance parameter when calculating the limit. The expected (median) limit on the branching fraction for $N_\mathrm{o} =N_\mathrm{b}(x_1)$ is $3.94\times 10^{-7}$ at 90 % CL. No events are observed in the signal region and the observed limit on the branching fraction is therefore $3.76\times 10^{-7}$ at 90 % CL.

## Conclusions and outlook

This article presents a search with the ATLAS detector for neutrinoless $\tau \rightarrow 3\mu$ decays using 20.3 fb$^{-1}$ of 2012 LHC *pp* collision data, utilising $\tau$ leptons produced in $W\rightarrow \tau \nu$ decays. No events are observed in the signal region for the final selection while $0.193 \pm 0.131_\mathrm{syst} \pm 0.037_\mathrm{stat}$ background events are expected. This results in an observed (expected) upper limit of $3.76\times 10^{-7}$ ($3.94\times 10^{-7}$) on $\mathrm{Br}(\tau \rightarrow 3\mu )$ at 90 % CL. Although this limit is not yet competitive with searches performed at *B*-factories [7, 8] and at LHCb [9], it demonstrates the potential of LHC data collected by ATLAS as a probe of lepton flavour violation in $\tau$ lepton decays. This analysis utilises single $\tau$ lepton production in an environment very different from *B*-factories, which rely on $\tau$ lepton pair production in $e^+e^-$ collisions. The method and sample presented here were used to improve the ATLAS muon trigger and reconstruction of low-$p_\mathrm{T}$, collimated muons relevant to the $\tau \rightarrow 3\mu$ search. The analysis is limited by the number of $W\rightarrow \tau \nu$ decays and by the systematic uncertainty, which depends on the size of the data sample. With the much larger data sets anticipated at Run 2 of the LHC, the sensitivity of ATLAS to lepton-flavour-violating decays will be improved significantly.

## Footnotes

1 ATLAS uses a right-handed coordinate system with its origin at the nominal interaction point (IP) in the centre of the detector and the *z*-axis along the beam pipe. The *x*-axis points from the IP to the centre of the LHC ring, and the *y*-axis points upwards. Cylindrical coordinates $(r,\phi )$ are used in the transverse plane, $\phi$ being the azimuthal angle around the *z*-axis. The pseudorapidity is defined in terms of the polar angle $\theta$ as $\eta = -\ln \tan (\theta /2)$. Angular distance is measured in units of $\Delta R \equiv \sqrt{(\Delta \eta )^{2} + (\Delta \phi )^{2}}$.
